# Chemical Exposomics in Human Plasma by Lipid Removal and Large-Volume Injection Gas Chromatography–High-Resolution Mass Spectrometry

**DOI:** 10.1021/acs.est.4c05942

**Published:** 2024-09-24

**Authors:** Hongyu Xie, Kalliroi Sdougkou, Bénilde Bonnefille, Stefano Papazian, Ingvar A. Bergdahl, Panu Rantakokko, Jonathan W. Martin

**Affiliations:** † Department of Environmental Science, Stockholm University, 106 91 Stockholm, Sweden; ‡ National Facility for Exposomics, Metabolomics Platform, Science for Life Laboratory, Stockholm University, 171 65 Solna, Sweden; § Department of Public Health and Clinical Medicine, Section for Sustainable Health, Umeå University, 901 87 Umeå, Sweden; ∥ Department of Public Health, Lifestyles and Living Environments Unit, National Institute for Health and Welfare, Neulaniementie 4, 702 10 Kuopio, Finland

**Keywords:** chemical exposome, blood plasma, molecular discovery, sample preparation, GC-HRMS, exposure

## Abstract

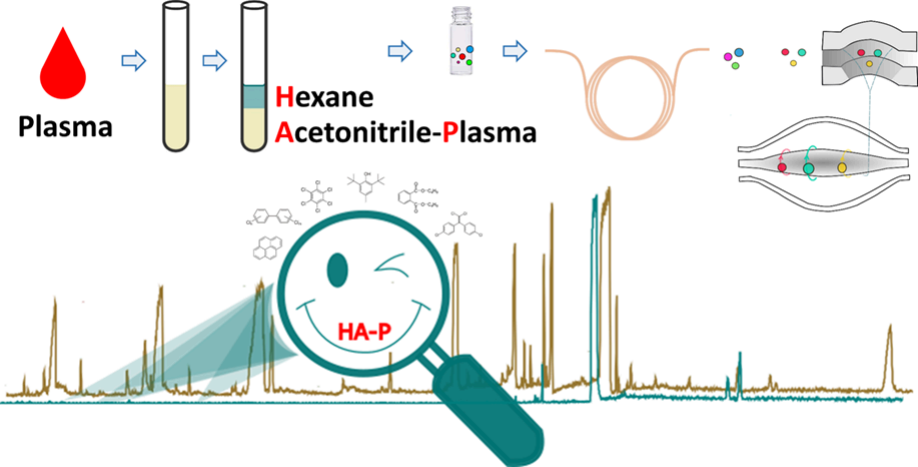

For comprehensive chemical exposomics in blood, analytical workflows are evolving through advances in sample preparation and instrumental methods. We hypothesized that gas chromatography–high-resolution mass spectrometry (GC-HRMS) workflows could be enhanced by minimizing lipid coextractives, thereby enabling larger injection volumes and lower matrix interference for improved target sensitivity and nontarget molecular discovery. A simple protocol was developed for small plasma volumes (100–200 μL) by using isohexane (H) to extract supernatants of acetonitrile-plasma (A-P). The HA-P method was quantitative for a wide range of hydrophobic multiclass target analytes (i.e., log *K*_ow_ > 3.0), and the extracts were free of major lipids, thereby enabling robust large-volume injections (LVIs; 25 μL) in long sequences (60–70 h, 70–80 injections) to a GC-Orbitrap HRMS. Without lipid removal, LVI was counterproductive because method sensitivity suffered from the abundant matrix signal, resulting in low ion injection times to the Orbitrap. The median method quantification limit was 0.09 ng/mL (range 0.005–4.83 ng/mL), and good accuracy was shown for a certified reference serum. Applying the method to plasma from a Swedish cohort (*n* = 32; 100 μL), 51 of 103 target analytes were detected. Simultaneous nontarget analysis resulted in 112 structural annotations (12.8% annotation rate), and Level 1 identification was achieved for 7 of 8 substances in follow-up confirmations. The HA-P method is potentially scalable for application in cohort studies and is also compatible with many liquid-chromatography-based exposomics workflows.

## Abstract 2

Sensitivity of chemical exposomics in plasma was enhanced after lipid removal and large-volume injections to GC-HRMS. Application to adult plasma resulted in high rates of target analyte detection, nontarget annotation, and molecular confirmation.

## Introduction

The concept of the exposome was introduced in 2005 to encourage more research on the environmental determinants of disease.^1,2^ Environmental chemicals have long been known to be human disease risk factors,^3^ and relevant exposures today include ambient air pollution and mixtures of contaminants ingested from food, water, and dust.^4−7^ Analytical methods are now evolving to measure the “chemical exposome”, broadly defined as all environmental chemical exposures throughout the life course.^8−10^ With this ambitious scope of chemical exposomics come several practical methodological challenges, including to comprehensively quantify a broad range of priority target analytes, to discover and identify novel exposures by nontarget workflows, to scale up sample throughput, and to achieve high sensitivity in the small volumes of biofluids available in typical cohort studies.^11^

Traditional analytical methods for human biomonitoring are mostly targeted and mass spectrometry-based, employing either liquid chromatography (LC) or gas chromatography (GC).^12−19^ Such methods are very sensitive and quantitative but can require large sample volumes and laborious sample processing steps and ultimately reveal only a limited fraction of the chemical exposome, defined *a priori* by the investigators. Today, there are over 350,000 chemicals in global commerce,^20^ but only 5% of these have ever been analyzed in environmental media,^21^ and likely fewer have been biomonitored in humans. In this context, modern instrumental advances in LC- and GC-high-resolution mass spectrometry (HRMS) provide great potential for chemical exposomics, as several commercial instruments now combine high mass spectral resolving power, high mass accuracy, high scanning frequency, good sensitivity, and a wide dynamic range in full scan mode.^10,22,23^ These instruments are therefore well suited to perform parallel target and nontarget data acquisition, but methodological challenges remain for sample preparation. One specific challenge is to quantitatively extract diverse analytes from complex biological samples while minimizing interferences. In human blood, known organic environmental contaminants vary greatly in their hydrophobicity, spanning 17 orders of magnitude in octanol–water partition coefficient (*K*_ow_), and their concentrations range over 11 orders of magnitude (i.e., 160 fM–140 mM)^24,25^ in a matrix dominated by major lipid classes and complex mixtures of endogenous metabolites^26^ that may overshadow small signals from environmental chemicals. Multiclass target exposomes are now being reported, as well as methods for combined target and nontarget analysis, but the sample preparation methods are often adapted directly from metabolomics,^27−29^ and not specifically designed or optimized for chemical exposomics, which strives to profile small molecules present at 1000× lower concentrations than endogenous substances.^25^

Most reported chemical exposomics methods for blood have so far been LC-based and thus focused on the polar environmental chemical fraction.^22,30,31^ However, the commercial availability of GC-Orbitrap HRMS instruments^32^ has enabled recent development of methods for low-polarity and semivolatile analytes.^29,33,34^ The associated sample preparation methods for blood serum and plasma are simple, rapid, and potentially scalable, including liquid–liquid extraction^29^ and liquid–liquid extraction with dispersive powders^34^ (i.e., QuEChERS). Nevertheless, the performance of existing methods to minimize major blood lipid coextractives has not been directly evaluated, and there is concern that abundant lipids may negatively influence method robustness, lower method sensitivity, and interfere with molecular discovery. Of particular relevance is that the resolving power of Orbitrap HRMS analyzers is adversely impacted by the abundant matrix signal, such that the auto gain control function dynamically lowers the ion injection time to minimize space-charging when an abundant signal is detected.^35,36^ Therefore, we hypothesized that by minimizing lipid coextractives, trace analytes would be more easily detected by GC-Orbitrap HRMS; moreover, larger volumes could be injected to improve method sensitivity.

Building on existing GC-based chemical exposomics methods for human blood, here, we explore methods to achieve improved sensitivity for plasma chemical exposomics by a GC-Orbitrap HRMS workflow. Specifically, we aimed to minimize coextracted plasma lipids at the extraction step to achieve dual benefits to method performance from (i) minimizing matrix interference and (ii) enabling large-volume injections (LVIs) of plasma extracts. A simple and potentially scalable protocol, which uses isohexane (H) to liquid–liquid extract acetonitrile-plasma (A-P), was developed and validated for small samples of human plasma (100–200 μL) while optimizing for lipid removal and method sensitivity for 103 priority target analytes. The validated method, termed the HA-P method, was applied to a subset of adult plasma samples (*n* = 32) in a combined target and nontarget analysis, and method performance was examined with respect to target analytes and molecular discoveries.

## Materials and Methods

### Chemicals, Standards, and Materials

A representative list of 103 target analytes from 6 chemical classes was selected based on higher detection frequencies in major historical biomonitoring initiatives (e.g., HBM4 EU, US NHANES), and also considering a broad range of physiochemical properties for use in method development, and their native and isotopic labeled standards were acquired from commercial suppliers (Table S1). These included 33 polychlorinated biphenyls (PCBs), 8 polybrominated diphenyl ethers (BDEs), 10 polychlorinated dibenzodioxins/dibenzofurans (PCDD/Fs), 16 polycyclic aromatic hydrocarbons (PAHs), 24 organochlorine pesticides (OCPs), and 12 phthalates. Toluene, formic acid, *n*-hexane, and silica gel (high-purity grade, pore size 60 Å, 230–400 mesh particle size) were from Merck (Germany). Isohexane, ethyl acetate, acetonitrile, dichloromethane, chloroform, and water were from Fisher Scientific (USA); isohexane was used instead of *n-*hexane because it has similar physiochemical properties but lower toxicity.^37,38^ MgSO_4_ and a dispersive solid-phase extraction powder, Bond Elut EMR-Lipid, were purchased from Agilent (USA).

### Human Plasma and Serum

For method development and validation, sterile-filtered human serum was obtained commercially (Merck, Germany, human male AB plasma-derived, origin USA). Standard reference material (SRM 1958, fortified and lyophilized human serum) was purchased from NIST (USA). The optimized exposomics workflow was applied to 32 individual human plasma samples (100 μL) from the Västerbotten Intervention Programme (VIP) cohort,^39^ a cardiovascular study cohort launched in 1985 in north Sweden. The samples (16 men and 16 women), which were collected between 1991 and 2013 (Table S2) and stored at −80 °C, were randomly selected among participants whose samples (separate aliquots) had previously been analyzed for persistent organic pollutants.^6^ Participant smoking and moisture snuff consumption as well as dietary intake of meat and fish were self-reported. As described previously,^31^ a Swedish pooled plasma reference sample was also prepared in-house from residual heparinized plasma of 953 adults (male and female) from the VIP cohort. Approval from the Swedish Ethical Review Authority [Dnr 2020-03301] was granted for work with these plasma samples.

### Sample Preparation of Human Plasma/Serum

All glassware was newly furnaced to minimize background contamination, and sample preparation was in a positive-pressure clean laboratory with high-efficiency particulate air filtration. Plastic containers and materials (e.g., disposable pipet tips) were avoided during sample preparation and analysis to minimize contamination of the samples and associated extracts. Method development included various tests of lipid removal techniques, including liquid–liquid and solid-phase extraction (see the Supporting Information, SI). The optimized method for human plasma and serum can be used for a range of small volumes typically available in cohort studies (100–200 μL). The plasma/serum was added to a conical borosilicate-glass centrifuge tube (10 mL, Pyrex, Corning, USA), and each sample was then spiked with 8 μL of a 100 ng/mL surrogate internal standard mixture containing 26 isotopically labeled chemicals (Table S1). For method development, small volumes of the internal standard solution were spiked onto each sample using precise Hamilton syringes (10 μL). For application to cohort plasma samples, the internal standard solution was indirectly added to larger volumes of acetonitrile and later added for protein precipitation using glass tip pipettes (200–2000 μL, Socorex). Procedural blanks composed of LC water, substituted for plasma, were prepared with all experiments and batches. For protein precipitation, a volume of acetonitrile was added that was 4 × sample volume (e.g., 400 μL of acetonitrile added to a 100 μL sample or 800 μL of acetonitrile added to a 200 μL sample), vortexed at 1400 rpm for 1 min (multitube vortex mixer, Ohaus, USA) and then centrifuged (5804R, Eppendorf) at 4400*g* for 5 min at room temperature. The supernatant was transferred to a new borosilicate-glass tube, and 1.2 mL of isohexane was added, followed by vortexing at 1400 rpm (1 min) and centrifugation at 3000*g* (1 min). The upper isohexane layer was transferred to a new borosilicate-glass tube; another 400 μL of isohexane was added to the extraction test tube, and the extraction step was repeated. The two isohexane layers were combined and evaporated to 100 μL by gentle nitrogen flow at room temperature (TurboVap LV, Biotage, Sweden), followed by adding 10 μL of 20 ng/mL volumetric internal standard (methoxychlor-D14) by a Hamilton syringe (700 series, 100 μL), vortexing, and transferring to an amber glass vial with an insert (0.3 mL, Thermo Scientific) for GC-HRMS analysis. All 32 individual samples (VIP cohort) were extracted in one daily batch along with 3 procedural blanks and 3 Swedish pooled plasma reference samples.

For comparison, plasma samples were also prepared by a leading literature chemical exposomics method designed for GC-Orbitrap HRMS.^29^ Briefly, 8 μL of 100 ng/mL surrogate internal standard was added to 200 μL of Swedish pooled plasma (*n* = 3) in conical glass tubes, followed by adding 50 μL of formic acid and 200 μL of *n*-hexane: ethyl acetate (v:v = 2:1). This was vortexed for 1 h on ice and then centrifuged at 4400*g* at 4 °C (10 min). The organic supernatant was transferred to a new tube with 25 mg of MgSO_4_, vortexed, and centrifuged at 4400*g* (10 min). The final supernatant was transferred to an injection vial with 6 μL of 100 ng/mL volumetric internal standard (methoxychlor-D14) for instrumental analysis.

### GC-HRMS Analysis

Plasma extracts were analyzed by GC (TRACE 1300 Series, Thermo Fisher Scientific, US) interfaced to an Orbitrap HRMS (Q-Exactive, Thermo Fisher Scientific, US) operating in full scan (34–750 *m*/*z*) in electron ionization mode. Nominal resolution was set to 60,000, with AGC Target at 1 × 10^6^, and Maximum IT set to auto. The Orbitrap was calibrated with the internal calibration gas and evaluated before every sequence, typically every 1–3 days. The ion source and transfer line temperature were 300 °C. The optimized method utilized a DB-5MS column (30 m × 0.25 mm × 0.25 μm, Agilent) and temperature gradient program starting at 30 °C for 1 min, increasing to 50 °C at 20 °C/min, then ramping to 170 °C at 25 °C/min, to 250 °C at 6 °C/min, and then increasing to 315 °C/min at 25 °C/min with a 12 min hold. The carrier gas was helium at a constant flow of 1.3 mL/min. Injections of 25 μL of extract were made to a programmable temperature vaporizer (PTV) injector with a baffle liner in large-volume mode (Table S3a). The temperature programs on the injection port and GC oven were coordinated. The starting temperature was held at 30 °C to evaporate the injection solvent (isohexane) while retaining the semivolatile analytes. During method development, a shorter DB-5MS column (15 m) was compared, based on instrumental detection limits (IDLs) of the target analytes, and 1 μL of each solution was injected into each system in PTV splitless mode, with injection temperature increasing from 30 to 315 °C at 7.3 °C/s, and oven and MS conditions were the same as in the optimized method. The IDL was defined as the lowest concentration in an 11-point calibration curve (range 0.0025–50 ng/mL, triplicate) with the signal-to-noise ratio >3, and at least 3 data points across the peak. For LVI, an optimal injection volume was decided by comparing responses for various injection conditions: 1 and 5 μL injections with a small syringe (10 μL) and 5, 10, 25, 30, and 40 μL injections with a larger syringe (100 μL); parameters for autosampler are shown in Table S3b. For investigation of lipid removal effects, the extracts prepared by the literature method were analyzed by the same instrumental method as above, but only with 2 and 5 μL injection volumes.

For application to cohort samples, calibration curves of target analytes were run 3 times during the injection sequence (beginning, middle, and end). Kovats retention index (RI) was applied, consisting of *n*-alkane standards (C7–40 mixture, Merck, 25 ng/mL in isohexane) injected separately at the beginning and end of the sequence. The entire sequence, including samples, calibration solutions, procedural and instrumental blanks, and Swedish pooled reference samples, lasted approximately 71 h (35 min gradient plus 15 min re-equilibration per injection), while all samples were maintained at 10 °C in the autosampler. The instrumental blanks consisted of a clean solvent and were run multiple times in the sequence to monitor for carryover.

### Method Validation

Method robustness was examined by 60 continuous injections of the same spiked (2 ng/mL) serum extract (split into 12 vials), with an additional 12 injections of isohexane spread throughout the sequence, with one solvent injection after every 5 sample injections. The whole sequence lasted 60 h, with no interruptions to the sequence. Target analyte recoveries, precision, and matrix effects were evaluated in triplicate spiked samples at 0.1, 1, and 10 ng/mL for all analytes in 200 μL of the commercial serum. An additional spike recovery experiment was also conducted at 75 ng/mL (*n* = 3) for those analytes with higher background concentrations. During method development, absolute recoveries (without internal standard correction) were calculated from the peak areas of native analytes spiked to serum before sample preparation compared to the same spike added to processed serum extracts immediately before GC-HRMS analysis. For method validation, final recoveries of spiked native analytes were calculated by the relative response to internal standards spiked before extraction. Method precision was evaluated by relative standard deviations of the triplicate recovery experiments at 1 ng/mL (if not available because of the native serum background, a value at a higher concentration was taken). For evaluating matrix effects, peak areas of spiked native analytes were compared between (i) extracts of serum and (ii) extracts of blank water. For analytes not present in method blanks, the method limit of quantification (MLOQ) was defined as the lowest concentration spiked to commercial serum (8 points between 0.005 and 10 ng/mL, *n* = 4), in which the peak area of the analytes had relative standard deviations below 20% with no internal standard correction but corrected for the volumetric internal standard. In the cases where background levels of the analytes in commercial serum interfered, the response of the corresponding isotopic labeled standard (spiked at 4 points between 0.005 and 5 ng/mL, *n* = 4) was used to calculate the MLOQ when possible; otherwise, solvent-based calibration curves were used (for 3 analytes). In the cases where the analyte was present in method blanks, the MLOQ was alternatively calculated from the average blank response plus 10 standard deviations. The linearity was evaluated by the *R*^2^ of standard solvent calibration curves (MLOQ–5 ng/mL, *n* = 3). Method accuracy was examined in two ways, including by comparison of calculated target analyte concentrations to a previous target analysis of the same VIP plasma samples (different aliquots)^6^ and by comparison of target analyte concentrations in NIST SRM 1958 with certified reference values.

### Data Processing and Analysis

External calibration curves (7 points, 0.005–5 ng/mL, *n* = 3) were used to quantify the 103 target analytes. TraceFinder (v.5.0, Thermo Scientific) was used for peak detection, identification, and quantification of the target analytes. Isotopically labeled surrogate internal standards (*n* = 26) were used to correct the recoveries and variations during sample preparation (Table S4). Target analytes were generally absent in procedural blanks; however, peaks were detectable for certain phthalates and PAHs, and in this case, analytes were only considered detected if sample peak areas were 3 times higher than the corresponding procedural blank; concentrations were also blank-subtracted in these cases.

For nontarget analysis, MS-DIAL^40^ (v.4.9.221218, parameters in Table S5) was used to align features and deconvolute corresponding electron ionization spectra. Each nontarget feature had a corresponding retention time (RT), RI, deconvoluted spectrum, and peak area. Mass spectral library matching was performed from the combination of an in-house GC-HRMS Orbitrap library (244 chemicals), MassBankEU,^41^ and NIST20 (version 2.4, purchased from Thermo Fisher Scientific, USA). Annotation confidence levels were applied and defined according to the annotation scoring framework for GC-HRMS,^42^ with RI matching scores considered.

Analyzed data were further processed and visualized in Excel (Microsoft Office 2019), Python (v.3.7.3),^43^ Jupyter Notebook (v.5.7.8),^44^ and R (v.4.3.2)^45^ and RStudio (v.2023.12.1 + 402)^46^ with ggplot2 (v.3.4.4).

Statistical tests were performed in R (v.4.4.1) using the rstatix package (v.0.7.2). Normality was evaluated by the Shapiro–Wilk test, equal variance was evaluated by the F-test (Data Analysis Tool, Excel), and group differences were evaluated by two-tailed *t* tests, with *p*-values adjusted for multiple testing by the Bonferroni method, if the data were normally distributed and had equal variance. Otherwise, statistical differences were evaluated by the two-tailed Wilcoxon–Mann–Whitney test, with the *p*-value adjusted by the Bonferroni method. For tests of correlation, the normality of the data was first tested by the Shapiro–Wilk method. Spearman's correlations were tested in R (stats package v.4.4.1).

## Results and Discussion

### GC Column Selection

In initial instrumental optimization, the IDLs of the 69 halogenated analytes (PCBs, BDEs, OCPs, and PCDD/Fs) were compared on two different GC column lengths (15 and 30 m; 0.25 mm × 0.25 μm DB-5MS, Agilent) using 1 μL injections of standard mixtures between 0.0025 and 50 ng/mL (Figure S1, original data in Table S6); phthalates and PAHs were not considered for this aim because of the low-level background contamination for certain analytes in these classes, making IDLs more difficult to determine on either column. For approximately half of the analytes (*n* = 35), the 30 m column resulted in better sensitivity (Figure S1, green), while for approximately one-third of the analytes (*n* = 22), the column length had no significant effect on detection limits (Figure S1, gray), and only 10 target analytes had better sensitivity on the shorter 15 m column (Figure S1, red); two analytes (pentachlorobenzene and PCB-3) had very low IDLs (i.e., ≪2.5 fg) that could not be adequately compared in the concentration ranges tested. Those analytes with lower detection limits on the 15 m column were mostly late-eluting (RI > 2570) substances with higher boiling points, including one OCP, five BDEs, and four PCDD/Fs. The highly brominated BDEs are known to degrade on longer or thicker GC columns and thus perform better on shorter columns.^47^ Nevertheless, for this multiclass chemical exposomics method, we decided to use the 30 m column because of the 2–5 fold increased sensitivity for a majority (51.5%) of the analytes examined and considering that this may lead to greater sensitivity for nontarget molecular discovery over most of the retention range.

### Optimization of Plasma/Serum Extraction

For further method development, commercial human serum was spiked with 103 priority target analytes (Table S4) representing the wide chemical space of hydrophobic environmental contaminants targeted by GC-based methodologies in national^48^ or international biomonitoring programs.^49^ These analytes belonged to 6 chemical classes (PCBs, BDEs, OCPs, PCDD/Fs, PAHs, and phthalates), ranged in molecular weight from 128 (i.e., naphthalene) to 637 (i.e., BDE-154) Da, and had log *K*_ow_ ranging from 1.7 (i.e., dimethyl phthalate) to 11.2 (i.e., dechlorane 603).^50^

For the traditional GC-MS sample preparation of blood plasma extracts, it is a common strategy for major lipid interferences to be removed by destructive techniques under acidic conditions, such as by addition of concentrated sulfuric acid^51,52^ or using chromatographic cleanup on acidified silica gel.^53^ For the suite of multiclass target analytes here, we briefly tested their recoveries in acidified silica gel columns by loading standards in isohexane (10–13.5 ng/mL). However, this step was relatively laborious and resulted in very low recoveries for a majority of PAHs and phthalates (<5%, Figure S2), likely due to degradation or hydrolysis under acidic conditions.^54,55^ To avoid these issues, this step was abandoned, and we focused on solvent extraction conditions that would minimize lipid coextractives. Acidic conditions were also avoided in further development; thus, prior to solvent extraction, we denatured and precipitated plasma proteins by addition of acetonitrile, rather than by formic acid.^29^ Plasma protein precipitation by acetonitrile is a common technique in metabolomics protocols and is also compatible with some LC-HRMS-based chemical exposomics methods,^31^ thereby opening the future possibility to split deproteinized supernatants for dual analysis by LC- and GC-based exposomics.

Following the protein precipitation and centrifugation step, further method development focused on minimizing lipid coextractives from the A-P supernatant. We first tested the effect of adding 40 mg of dispersive solid-phase extraction material (Bond Elut EMR-Lipid, preconditioned with 250 μL of water) on relative recoveries into isohexane extracts (detailed in the SI). Although EMR-Lipid reduced major sterol lipids in the chromatograms (Figure S3a, RT = 24.5 min), 25 late-eluting analytes (i.e., RT > 20 min) were also lost (average 20% absolute loss, range 10–28%). Moreover, the precision was much lower using EMR, and few improvements to the relative recoveries were evident (Figure S3b). Subsequent tests focused solely on solvent types and their ratios under liquid–liquid extraction conditions, including pure isohexane and mixtures of isohexane with more polar solvents (isohexane: toluene (9:1); isohexane: ethyl acetate (2:1); isohexane: chloroform (9:1)) (Figure S4). However, the addition of polar solvents always resulted in decreased recoveries, in particular with ethyl acetate, and especially for the more polar early-eluting analytes, likely because the tested polar solvents partition significantly into the acetonitrile phase. Moreover, the addition of toluene resulted in three layers and was abandoned. In all cases, the isohexane-containing extract layer (on the top) also contained more interference peaks of lipids or fatty acids when polar solvents were included.

Therefore, it was concluded that 100% isohexane (H) was the optimum extraction solvent for the A-P supernatant; in subsequent sections, we refer to this as the HA-P method. It is germane for us to note that a recent study investigating various extraction conditions for plasma exposomics reported the best quantitative performance for a hexane-acetonitrile-plasma extraction condition, but the authors chose alternate methods due to the assumption that the hexane layer would contain lipid interferences;^56^ as discussed later, the hexane layer in the HA-P method was in fact free from most lipid interferences.

In final HA-P method optimization, we compared target analyte recoveries with different volumes of isohexane for the primary extraction (600 μL, 1.2, and 2 mL). Although the larger volume (2 mL) slightly improved recovery for some analytes (average 3%), precision was lower (i.e., see higher standard deviations in Figure S5a). Therefore, 1.2 mL was determined to be the optimum volume of isohexane. Moreover, we tested the effect of performing a second follow-up extraction with the addition of isohexane. The addition of this secondary isohexane extraction (400 μL) after the primary extraction (0.6 mL) increased the absolute recoveries on average by 8.5% (range 1.1–14.4%, Figure S5b), particularly for later-eluting analytes. Thus, this method was included in the optimized HA-P method for validation tests.

### Performance of LVI

In order to increase the sensitivity for trace levels of contaminants, an LVI method was applied and optimized. The initial parameters (temperature gradient ramp, split flow rates, etc.) were first optimized with pure standards (Table S4), and the optimal injection volume was selected based on performance with a commercial serum extract, prepared by the optimized HA-P method and spiked at 10 ng/mL. Peak areas increased with larger injection volumes between 1 and 40 μL (Figure S6), but at 30 and 40 μL, the peak areas did not further increase linearly, and some analytes had greater standard deviations and split peaks, suggesting overloading at these injection volumes. Thus, 25 μL injections were chosen as optimal for method validation tests. Under these conditions, method precision and robustness were tested over 4 days by 60 continuous injections of the same spiked sample extract (2 ng/mL). Although peak areas for many target analytes decreased slowly over the entire sequence (mean of 28%), this minor effect was adequately controlled by internal standard correction. The median RSD was 5.1% for target analytes over 4 days (range 1.6–24.5%, Figure S7).

### Method Validation

**Figure 1:**
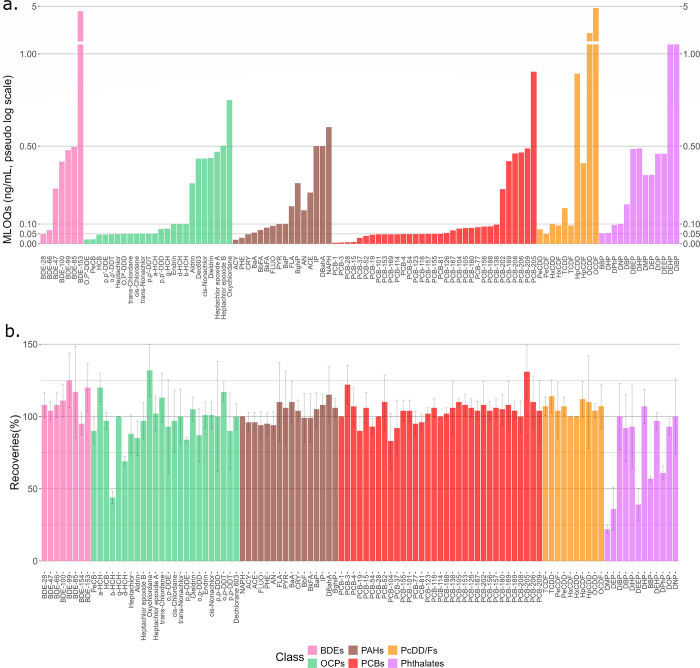
Method validation results for multiclass target analytes, arranged by classes, spiked to 200 μL of human serum and analyzed by the HA-P method with LVI, showing (a) MLOQ (*n* = 4) and (b) internal standard corrected analyte recoveries (*n* = 3, 1 ng/mL spiking levels). Panel (a): MLOQ of BDE-154 was >10 ng/mL and is not plotted. Panel (b): 13 analyte recoveries were calculated at 10 or 75 ng/mL (detailed in Table S7).

Using the optimized HA-P method with LVI (25 μL), we performed method validation for all target analytes, including determination of matrix effects, internal standard corrected recoveries, calibration linearity, and MLOQs (Table S7). The internal standard for correcting each native analyte was selected based on similar retention, absolute recovery, and matrix effect. The median MLOQ was 0.088 ng/mL (range 0.005–4.83 ng/mL; Figure 1a), and for most analytes (99 out of 103), calibration curve linearity was good (*R*^2^ > 0.99) between the MLOQ and 5 ng/mL. Two phthalates (diethyl phthalate and dibutyl phthalate) had relatively lower *R*^2^ values (0.98 each), and diisobutyl phthalate and di(2-ethylhexyl) phthalate were semiquantified by single-point calibration because of background interference at lower contamination (detailed in Table S7). When all analytes were spiked to native serum at 1 ng/mL, among the 91 detectable analytes (out of 103), the mean matrix effect was null (i.e., mean 100% response; range 76–140%) and the median internal standard recovery was 104% (range 22–132%; Figure 1b). For 22 analytes with interfering background levels in method blanks or commercial serum, spiking experiments at 10 or 75 ng/mL showed a mean recovery of 96% and a median matrix effect of 110% (Table S7). The lowest recoveries (22–41%) and worst matrix effect (513%) were found for three phthalates, specifically dimethyl phthalate, diethyl phthalate, and diethoxyethyl phthalate, likely owing to their lower hydrophobicity (log *K*_ow_ = 1.6, 2.5, and 2.1)^57^ and their presumed preferential partitioning to the plasma-acetonitrile layer during extraction. All other spiked target analytes have log *K*_ow_ > 3; thus, we suggest that the HA-P method is only appropriate for analytes in this more hydrophobic range. This limitation of the HA-P method is not a limitation for chemical exposomics in general, for example, dimethyl phthalate and diethyl phthalate are more sensitive and quantitative by LC-HRMS-based chemical exposomics,^58^ and it is understood that GC-HRMS will need to be applied together with LC-HRMS for comprehensive analysis of the exposome.^9^ Finally, it is noteworthy that the relatively low recovery of β-HCH and relatively high recovery of α-HCH (Figure 1b) are likely due to interconversion of these isomers in the presence of water (i.e., plasma)^59^ and therefore not necessarily a limitation of the analytical method.

The performance of the HA-P method with LVI was also examined by analysis of NIST SRM 1958 (i.e., a freeze-dried fortified human serum), which has certified or noncertified reference values, for 33 of the analytes targeted here. For most of these (*n* = 23), quantified concentrations by the HA-P method were within 75–125% of certified/noncertified values (i.e., ratio 0.75–1.25, Figure S8), and the mean ratio for all analytes was 0.98 (range 0.32–1.43). Notable outliers were again HCH isomers (i.e., low concentrations of β-HCH and high concentrations of γ-HCH), which have noncertified values in the SRM and are known to interconvert as described above. The authors of the literature method reported that the ratios of 20 target analytes were within 75–125% of certified/noncertified values and that the mean ratio for all analytes was 0.9 (range 0.3–1.79).^29^ Overall, the current chemical exposomics method performs similarly as well as the literature chemical exposomics method for target analysis based on analysis of the same SRM (see Figure S8 for comparison).^29^

### Investigation of Plasma Lipid Interferences

During method development, it was noted that the total ion chromatograms of HA-P method extracts were relatively clean (visually) and free from large interfering peaks in GC-HRMS analysis (Figure S3a). This suggested that few lipid species had been coextracted and may explain why dispersive solid-phase extraction by EMR-Lipid had no beneficial effect in our method development tests. The major lipids in human plasma include glycerolipids, glycerophospholipids, and sterol lipids such as cholesterol esters.^26^ Nonpolar bulk lipids (i.e., glycerolipids and some sterol esters) generally have very low solubility in polar solvents such as acetonitrile and are known to be removed with proteins during protein precipitation.^60^ Most phospholipids remain in the plasma-acetonitrile phase during extraction, and here, we found no traces of phospholipids in the isohexane extract (Figure S9, cyan).

**Figure 2:**
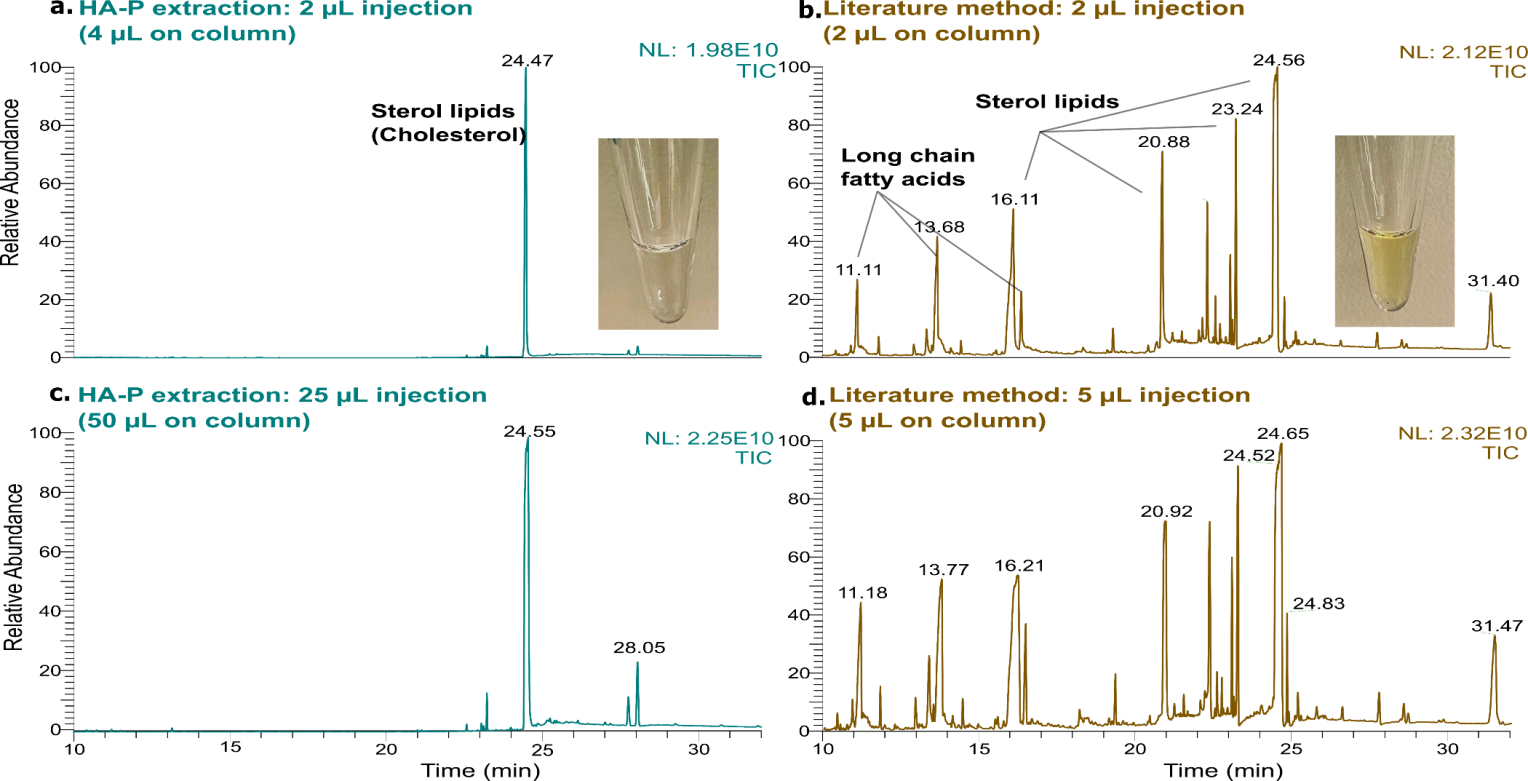
Total ion chromatograms and photos of extracts from pooled Swedish plasma (200 μL, *n* = 3 each) prepared by the HA-P method (cyan, left panels) and a literature method (brown, right panels) injected with various volumes to GC-HRMS. Chromatograms for the HA-P method extract are shown for a 2 μL injection (a) and a 25 μL injection (c), corresponding to 4 and 50 μL of plasma equivalents on-column, respectively, and these can be contrasted with chromatograms for the literature method extract for a 2 μL injection (b) and a 5 μL injection (d), corresponding to 2 and 5 μL of plasma equivalents on-column. Photos of the associated solvent extracts are also shown for (a) HA-P method in 100 μL of isohexane and (b) from the literature method in 200 μL of ethyl acetate, with 100 μL taken for photography.

To fully understand the relative extent of lipid interferences, and the impact of these on chemical exposomics, we compared plasma extracts from the HA-P method (in 100 μL of isohexane) to extracts of the same plasma by a literature method (in 200 μL of ethyl acetate).^29^ The relatively clear appearance of extracts from the HA-P method was evident, relative to yellow-colored extracts by the literature method (Figure 2). Consistent with visual appearances, after injecting 2 μL of each extract (Figure 2a,b), the corresponding total ion chromatograms revealed a comparably complex matrix for the literature method, with many abundant coextracted substances. The relatively clean total ion chromatogram of the HA-P extract is noteworthy considering that twice as much plasma equivalents were injected on-column. The major chromatographic peaks for the literature method extract included long-chain fatty acids (RT = 11.19, 13.77, 16.46 min) and sterol lipids (RT = 16.06, 20.96–24.61 min, Figure S10b), which were either absent or much lower in the HA-P method, even with 10-fold more plasma equivalents injected on-column (Figure S10a).

For the extracts from the literature method, the MS response was saturated for many of the largest peaks, as maximum peak heights were in the range of 2 × 10^10^ and did not increase with increasing injection volumes from 2 to 5 μL. The largest peaks in TICs of both extracts were for sterol lipids (RT = 24.5 min); thus, both methods are still similarly prone to interference from these major plasma metabolites. When the injection volume of the plasma extract was increased from the literature method (Figure 2d), the major chromatographic peaks became visibly broader, suggesting that the GC column was overloaded, and larger injection volumes were not attempted. In comparison, a larger injection volume of the HA-P extract (25 μL, corresponding to 50 μL plasma equivalents on-column, Figure 2c) was nevertheless applied, and the total ion chromatogram was still relatively clean but did show elevated baseline at later retention times (>25 min). This increased background overlaps with the retention range of 18 (17.5%) of the target analytes; thus, its potential as a minor interference cannot be discounted.

**Figure 3:**
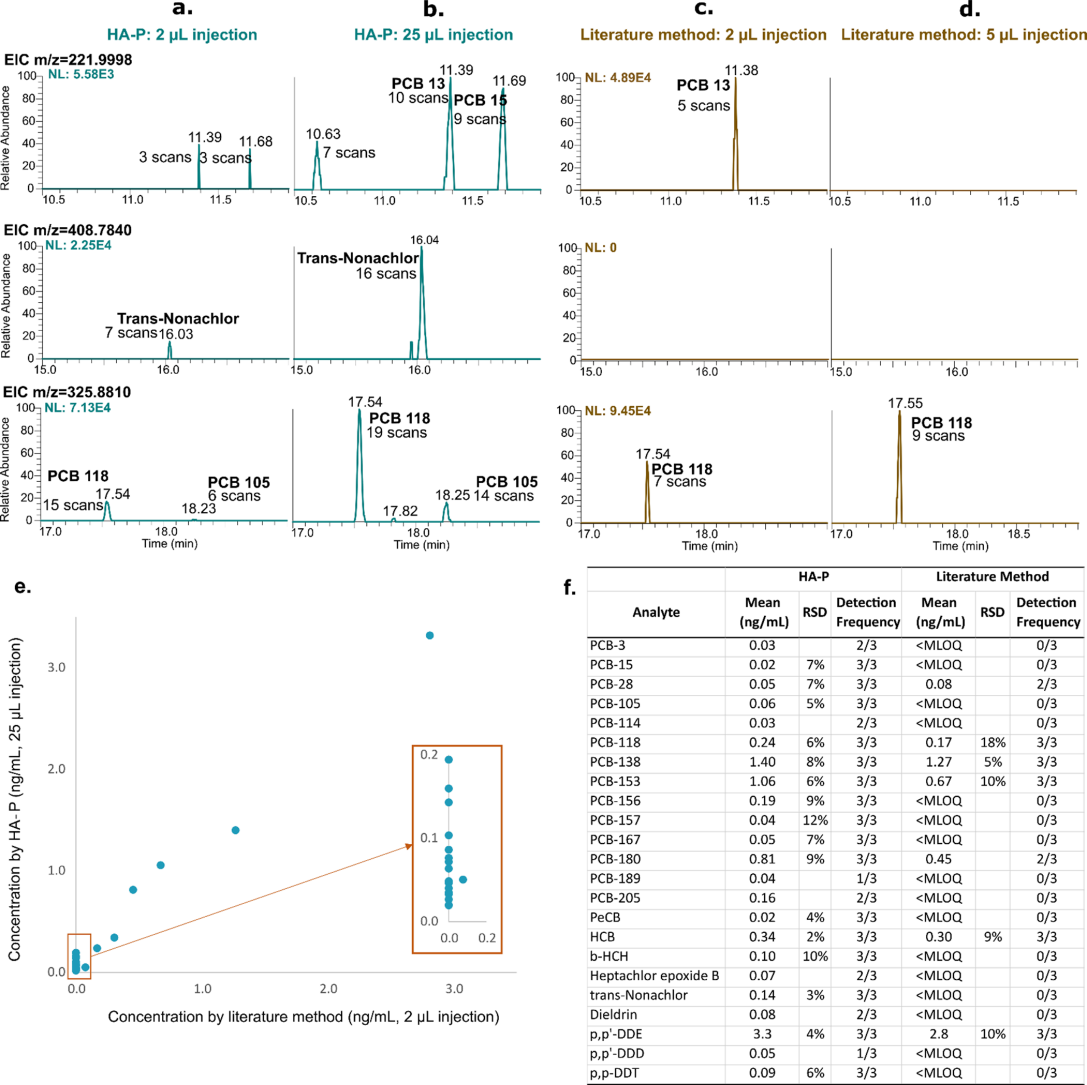
Example extracted ion chromatograms (EICs, a–d), as well as concentrations and detection frequencies (e, f) of analytes in triplicate extracts of pooled Swedish plasma (200 μL aliquots) prepared by the HA-P method and literature method and injected to GC-HRMS with various injection volumes. Panels (a) and (b) present the HA-P method and injection volumes of 2 and 25 μL. Panels (c) and (d) present the literature method and injection volumes of 2 and 5 μL, respectively. [Top row (panels a–d) is the EIC of ion *m*/*z* 221.9998 for PCB 13 (Level 4 annotation, RT = 11.39 min), PCB-15 (RT = 11.89 min), and other dechlorinated PCBs. Middle row (panels a–d) is the EIC of *m*/*z* 408.7840, corresponding to trans-nonachlor (RT = 16.03 min). Bottom row (panels a–d) is the EIC of ion *m*/*z* 325.8810 for PCB-118 (RT = 17.54 min) and PCB-105 (RT = 18.25 min).] Panel (e) shows general agreement of quantified targeted analyte concentrations by the two methods above 0.2 ng/mL, but below this approximate threshold, the literature method resulted in nondetection for most analytes; mean concentrations and detection frequencies are listed in panel (f).

As shown in extracted ion chromatograms of example target analytes in the HA-P method (Figure 3a,b), the increased injection volume resulted in larger target analyte peaks and also revealed new detectable analyte peaks that were not evident at lower injection volumes (e.g., trans-nonachlor, *m*/*z* 408.7840 at 16.04 min, and PCB-105, *m*/*z* 325.8810 at 18.25 min), demonstrating enhanced method sensitivity by LVI for low-abundance analytes in plasma. Using 200 μL plasma aliquots of pooled Swedish plasma, 16 target PCBs/OCPs (0.02–3.3 ng/mL) were consistently detected in triplicate (*n* = 3/3) samples by the HA-P method (25 μL injection), compared to only 5 target analytes (0.17–2.8 ng/mL) by the literature sample preparation method (2 μL injection) and the same instrumental settings (Figure 3e,f). Among the 11 PCBs/OCPs not consistently detected by the literature method, most had plasma concentrations below 0.2 ng/mL, according to the HA-P method, except for PCB-180 (0.45 ng/mL), which was inconsistently detected (2 of 3 replicates) by the literature method, despite relatively higher concentrations.

All target analytes detected and quantified in SRM 1958 (*n* = 33 analytes) had similar accuracies by the HA-P method and literature method, including those analytes present at moderate to high concentrations (i.e., 293–1250 ng/kg, certified values) and at lower concentrations, such as for PCB-123 and PCB-114 (0.0525, 0.0466 ng/kg, noncertified values) (Figure S8). When analyzing the pooled Swedish plasma in triplicate by the HA-P and literature methods, the detection and quantification were similar by both methods when concentrations were above 0.2 ng/mL (Figure 3e,f). The major discrepancies were at lower concentrations, where most analytes were nondetectable by the literature method, thereby demonstrating enhanced sensitivity by the HA-P extraction method and LVI.

**Figure 4:**
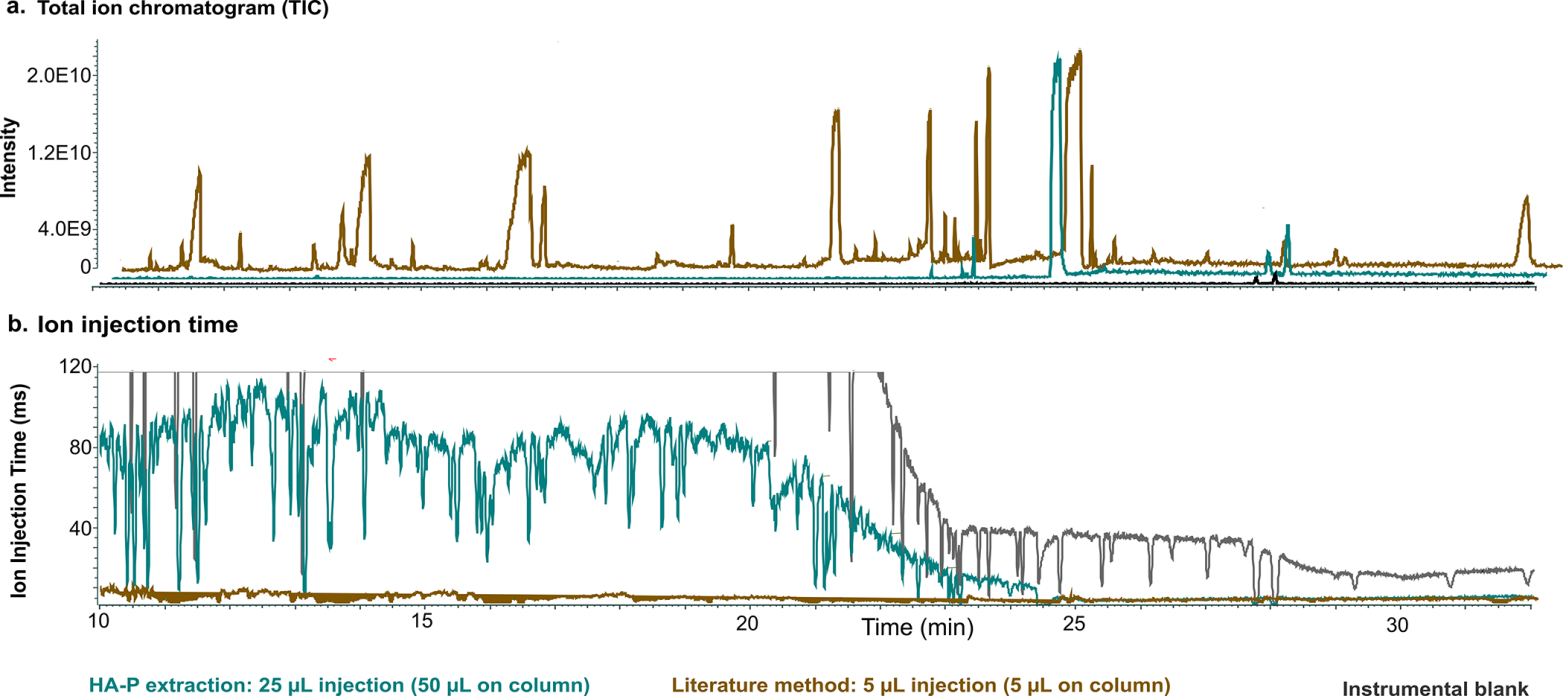
Comparison of (a) total ion chromatograms and (b) ion injection times for extracts of pooled Swedish plasma prepared by the HA-P method (cyan, 50 μL plasma equivalents on-column) and by a literature method (brown, 5 μL plasma equivalents on-column), both injected to the same GC-HRMS. In both plots, the instrumental blank is also shown (black, 25 μL isohexane) for comparison.

An additional observation in the analysis of the extracts produced by the literature method was that, when increasing injection volumes from 2 to 5 μL, some analyte peaks disappeared (e.g., PCB-13 at 11.39 min, *m*/*z* 221.9998) (Figure 3c,d). This may be due to abundant interferences (Figure 4a), and the auto gain control function of the Orbitrap mass spectrometer, which applies a dynamic ion injection time (i.e., to the C-trap and subsequently to the Orbitrap analyzer) throughout the analytical run to balance sensitivity and mass spectral resolving power.^35,36^ For a very clean injection, as shown for analysis of instrumental blanks composed only of isohexane (Figure 4b, black), the ion injection time was initially maximal (i.e., 112 ms), thereby allowing maximum signal to the Orbitrap analyzer, but declined to approximately 20 ms after 22 min due to increasing background signal from column-bleed at higher temperatures (300 °C at 22 min); overall, the mean ion injection time throughout the analysis of isohexane instrumental blanks was high (mean = 73 ms; 97 ms before 25 min). In comparison, lower ion injection times were observed with LVIs of the HA-P extract (mean: 38.6 ms, 56 ms before 25 min, Figure 4b, cyan). Nevertheless, these ion injection times were still substantially higher than that for the extract produced by the literature method (mean: 3.22 ms, 3.85 before 25 min, Figure 4b, brown), even considering a 10× more plasma-equivalent volume injected on-column from the HA-P extract (Figure 4b; 50 μL plasma equivalents by HA-P (25 μL injection) and 5 μL plasma equivalents by the literature method (5 μL injection)). Lower ion injection times predictably corresponded to regions of the chromatograms with a higher total ion signal (Figure 4a). For analytes that are still detectable, a correction factor is applied by the software to maintain quantitative analysis,^61^ but for trace analytes near detection limits, the signal can become nondetectable due to the lower ion injection time, as shown for PCB-13 in the extract from the literature method (Figure 3d).

### Multiclass Target Analysis of Individual Plasma Samples

**Figure 5:**
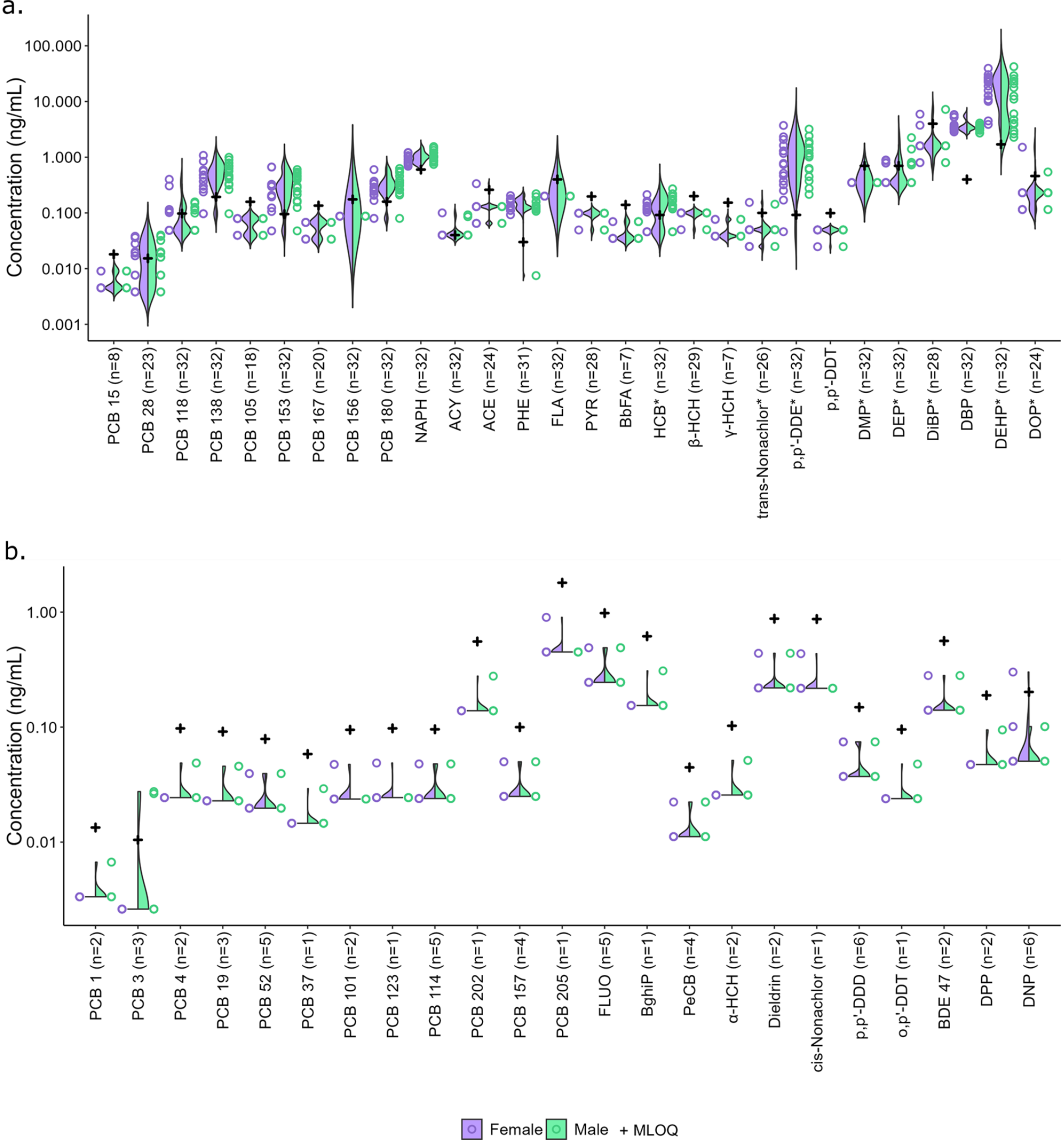
Violin plots showing concentrations and distributions for detected target analytes in 32 individual Swedish adult plasma samples by sex (female in purple, male in green). Panel (a) shows 28 analytes with detection frequencies >20%, while panel (b) shows 23 analytes detected at lower frequencies (i.e., in 1–6 samples). Black “+” symbols indicate the target analyte MLOQ. For data visualization, detectable signals below the MLOQ are plotted as MLOQ/2, and nondetectable signals are plotted as MLOQ/4. For analytes marked with black “*” symbols, the MLOQs were calculated by methods other than the native standard in the matrix-matched calibration curve, detailed in Table S7. Values of DBP were extrapolated from the calibration curves, and DiBP and DEHP were semiquantified using one point.

The HA-P method with LVI was applied to 32 individual plasma samples of Swedish adults as well as to pooled Swedish plasma for reference and quality assurance. Among all samples, 51 (out of 103) target analytes were detected in at least one individual (Table S8). The detected analytes included 7 dioxin-like PCBs (#105, #114, #118, #123, #156, #157, and #167), 14 nondioxin-like PCBs (#1, #3, #4, #19, #15, #28, #52, #37, #101, #138, #153, #202, #180, and #205), 9 PAHs, 12 OCPs, 1 BDE, and 8 phthalates. Among these, 28 analytes had detection frequencies above 20%, the distributions of which are shown by sex (Figure 5), and 9 analytes (β-HCH, DEHP, PCB-156, pyrene, p,p’-DDT, DiBP, PCB-167, HCB, and acenaphthene) showed gender differences (*p* < 0.05), but they were not significant anymore after the Bonferroni correction. Contrasting the current results to previous target analyses of the same plasma samples (separate aliquots)^6^ showed linear associations between the two methods (Figure S11).

**Figure 6:**
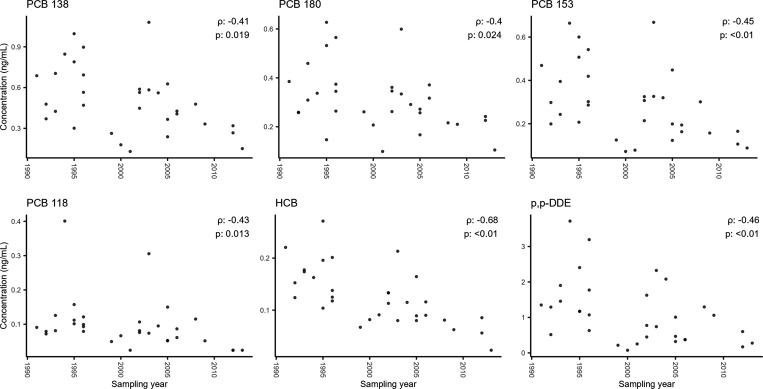
Concentrations of example target analytes, with statistically significant negative associations with sampling year; the Spearman correlation coefficient (ρ) and statistical significance (*p*) are shown in each plot.

Correlations between concentration and sampling year were tested by the Spearman method as most data were not normally distributed. Twelve target analytes showed statistically significant temporal associations between 1990 and 2013 (test method and statistics in Table S8), six of which are shown in Figure 6. Particularly, PCBs and OCPs were negatively associated with sampling year, consistent with bans and restrictions that started in the 1970s.^62,63^ To the contrary, DEHP, a commonly used phthalate plasticizer, showed a positive association with sampling year, from below 10 ng/L in the early 1990s to >40 ng/L by 2005, and possibly lower concentrations thereafter. We acknowledge that no field blanks were available in this cohort to rule out phthalate contamination from medical sampling equipment, but similar or higher levels of DEHP have been reported in other studies. For example, mean DEHP was 180 ng/mL (maximum 1030 ng/mL) in healthy women in the 2000s^64^ and 65 ng/mL in the serum of women diagnosed with endometriosis in the 2010s.^65^

We found no significant associations between the concentrations of these target analytes and individual metadata such as birth year, sampling age, meat consumption (Spearman correlation test), or smoking status (two-tailed Wilcoxon mean value test). Nevertheless, concentrations of several PCBs (#15, #156, and #180), as well as β-HCH and HCB, were significantly higher in snuff users than nonsnuff users (means: 0.008 vs 0.002 (*p* < 0.01), 0.07 vs 0.05 (*p* = 0.02), 0.37 vs 0.05 (*p* = 0.02), 0.06 vs 0.05 (*p* = 0.03), and 0.15 vs 0.11 ng/mL (*p* = 0.05), respectively, for PCB-15, 156, 180, β-HCH, and HCB), whereas DEHP was lower in snuff users (mean: 8.5 vs 21.4 ng/mL; *p* < 0.01) by the two-tailed Wilcoxon mean value test. However, snuff users were not equally distributed over sampling years (with fewer snuff users in later years); thus, these results are likely confounded by the associated temporal trends.

### Nontarget Analysis of Individual Plasma Samples

We additionally evaluated the suitability of the HA-P method with LVI for the discovery of unexpected substances in a nontarget exposomics workflow. After data processing by MS-DIAL and blank filtration, a total of 875 molecular features were detectable among all individual plasma samples (see Table S9, including relative responses). Among these features, 112 were matched to reference library spectra, corresponding to a relatively high annotation rate of 12.8%. The annotations included 30 of the target analytes (confirmed Level 1,^42^ ΔRT < 1%), as well as 82 new annotations (Level 2 confidence,^42^ ΔRI < 50) that included 28 prospective environmental substances. Authentic standards for 8 of these environmental chemicals were purchased, resulting in 7 confirmed identifications (Level 1, ΔRT < 1%, chromatograms and spectra in Figures S12–18). These included the related analytes 2,4-di-*tert*-butylphenol (Figure S12) and tris(2,4-di-*tert*-butylphenyl) phosphite (Figure S18), which have been used as antioxidants and UV stabilizers in rubber and plastics; the co-occurrence of the two was reported in indoor dust in 2018.^66^ Although 2,4-di-*tert*-butylphenol, which is a degradation product of the latter^67^ and other substances, has been detected in human blood and urine previously,^68,69^ we are not aware that tris(2,4-di-*tert*-butylphenyl) phosphite has been reported previously in human biomonitoring. This latter substance was previously reported in chemical migration tests from polymeric materials to water and simulated foods.^66,67,70,71^ This analyte has a high boiling point and very late elution time in our method (26.14 min) and in some methods may suffer from high background interferences. The discovery of this substance and, in general, the high nontarget annotation and confirmation rates are likely due to a combination of compounding factors, including higher sensitivity from LVI and lower matrix interference in the HA-P extracts. The low matrix interference by the HA-P method may not only result in higher sensitivity (i.e., due to higher ion injection times) but could also improve the performance of the *in silico* spectral deconvolution (i.e., in MS-DIAL), resulting in higher quality spectra that will have better matches to spectral libraries.

In this study, a comprehensive multiclass target and nontarget chemical exposomics method was developed and validated for human plasma. With the combination of lipid removal at the extraction stage and LVI, the method sensitivity was significantly improved in comparison to a literature GC-HRMS method without these steps. These developments resulted in more target analytes being consistently detected and a higher rate of molecular annotation and confirmation in nontarget analysis. An important reason for the enhanced method performance was the longer ion injection times achieved in the Orbitrap instrument throughout the GC run time owing to a much lower signal from coextracted lipids. The method was rugged and can be applied to larger studies of the chemical exposome in combination with LC-HRMS-based analysis.

## Supporting Information Available

Methods; IDL for multiclass target analytes on a 15 and 30 m DB-5MS column; absolute recoveries during method development; increased peak areas with injection volumes; responses of targeted analytes during 60 continuous 25 μL injections; quantified values by two compared methods of the SRMs; EIC of the phosphorylcholine fragment ion; extracted ion chromatograms of lipids in Swedish plasma by two compared methods; quantified values by two methods of the same sample; and confirmed nontarget analytes (PDF)
Native and labeled standards; cohort sample distribution; PTV and autosampler method; information of multiclass target analytes and IS; MS-DIAL parameters; IDLs; validation results; target results; and nontarget results (XLSX)
