# Supplementary material for: Chemical Exposomics in Human Plasma by Lipid Removal and Large-Volume Injection Gas Chromatography–High-Resolution Mass Spectrometry
Source: Environ Sci Technol. 2024 Sep 24;58(40):17592–605. doi: 10.1021/acs.est.4c05942 (PMC11465644; doi:10.1021/acs.est.4c05942)
Supplement: Supplementary file 1 — es4c05942_si_001.pdf [file es4c05942_si_001.pdf]

Supplementary Information To:

Chemical Exposomics in Human Plasma by Lipid Removal and Large Volume Injection Gas Chromatography High-Resolution Mass Spectrometry

Hongyu Xie[1], Kalliroi Sdougkou[1], Bénilde Bonnefille[1,2], Stefano Papazian[1,2], Ingvar A. Bergdahl[3], Panu Rantakokko[4], Jonathan W. Martin[1,2]*

[1]Department of Environmental Science, Stockholm University, Stockholm 106 91, Sweden

[2]National Facility for Exposomics, Metabolomics Platform, Science for Life Laboratory, Stockholm University, Solna 171 65, Sweden

[3]Department of Public Health and Clinical Medicine, Section for Sustainable Health, Umeå University, Umeå 901 87, Sweden

[4]National Institute for Health and Welfare, Department of Public Health, Lifestyles and Living Environments Unit, Neulaniementie 4, Kuopio 702 10, Finland

Corresponding author: Jonathan W. Martin; Email: jon.martin@aces.su.se

Summary: 27 pages, 18 figures, and 10 tables in a separate excel file.

Supplementary Methods p. S3

Supplementary Figures p. S4 -S27

Figure S1. Ratio of instrumental detection limits for multi-class target analytes analyzed on a 15 m and 30 m DB-5MS column
Figure S2. Absolute recoveries of targeted analytes in isohexane (10 ng/mL) cleaned up by acidic silica gel column
Figure S3. Chromatograms and relative recoveries of targeted analytes from spiked commercial serum prepared with and without additional lipid cleanup step by Bond Elut EMR-lipid
Figure S4. Relative recoveries of targeted analytes from spiked commercial serum extracted by pure isohexane or isohexane mixed with polar solvent
Figure S5. Recoveries of targeted analytes from 200 µL spiked serum extracted with various volumes of isohexane
Figure S6. Increased peak areas of multi-class target analytes with injection volumes of the same spiked serum extracts
Figure S7. Responses of targeted analytes during 60 continuous 25 µL injections of spiked serum extract
Figure S8. Quantified values by HA-P method and literature method of the standard reference materials
Figure S9. Extracted ion chromatograms of phosphorylcholine fragment ion (common fragment of phospholipids)
Figure S10. Extracted ion chromatograms of long chain fatty acids and sterol lipids extracted from pooled Swedish plasma when prepared by literature method and HA-P method
Figure S11. Quantified values by HA-P method and previous target studies of the same (different aliquots) samples
Figure S12. Extracted ion chromatogram and full scan spectrum for 2,4-di-tert-butylphenol
Figure S13. Extracted ion chromatogram and full scan spectrum for butylated hydroxytoluene
Figure S14. Extracted ion chromatogram and full scan spectrum for tributyl phosphate
Figure S15. Extracted ion chromatogram and full scan spectrum for hexadecanoic acid, methyl ester (Methyl palmitate)

Figure S16. Extracted ion chromatogram and full scan spectrum for methyl-3-[3,5-di(tert-butyl)-4-hydroxyphenyl] propanoate (metilox)
Figure S17. Extracted ion chromatogram and full scan spectrum for 2,4-bis(1-methyl-1-phenylethyl) phenol (2,4-bis(2-phenylpropan-2-yl) phenol)
Figure S18. Extracted ion chromatogram and full scan spectrum for tris(2,4-di-tert-butylphenyl) phosphite (Phosphite 168)

Supplementary Tables
Table S1. Native and isotopic labelled chemical standard list
Table S2. Distribution of sampling year and age information of the analyzed 32 individuals' sample
Table S3a. PTV program parameters
Table S3b. Autosampler parameters
Table S4. Target analytes and internal standards
Table S5. MS-DIAL nontarget data analysis parameters
Table S6. Instrumental detection limits of targeted analytes on 30 and 15 m columns
Table S7. Validation results for multi-class target analytes
Table S8. Target analytes detected and quantified in authentic human plasma samples (n=32) from the Västerbotten Intervention Programme (VIP) cohort
Table S9. Nontarget features and their relative peak areas in authentic human plasma samples (n=32) from the VIP cohort.
(separate file; XSLX)

Supplementary Dataset

MS datasets of pooled samples have been deposited as mzXML files at the MassIVE database with the identifications: MSV000094928

Sample preparation methods with acidic silica gel or Bond Elut EMR-lipid

In order to have clean matrix to support large volume injection, method development of this protocol included tests of other lipid removal steps, including liquid-liquid and solid-phase extractions. Solid-phase extractions methods with acidic silica gel cleanup and Bond Elut EMR-lipid (Agilent, USA), detailed explained as below.

Clean up test with acidic silica gel. Acidic silica gel was prepared with sulfuric acid adjusted from methods prescribed by the US CDC NHANES.[1] From bottom up, cartridges were packed with glass wool, 0.1 g activated silica gel, 1 g silica gel mixed with 33% sulfuric acid, and 0.25 g activated silica gel. Two types of cartridges were used, SPE cartridge (Supelco, USA) and glass pasteur pipets (26 cm long, Fisher Scientific, USA), each in duplicate samples. Native standard mixture (10–13.5 ng/mL) in isohexane was gently loaded to the top of the packed column, then eluted with 20 mL 10% dichloromethane in isohexane. The eluate was collected and evaporated to 0.5 mL, and 1 µL was injected to the GC-HRMS. The recoveries of PAHs and phthalates were low (<5%, **Figure S2**), likely due to due to degradation under the acidic conditions.

EMR lipid removal. For 200 µL of spiked serum, after protein precipitation with acetonitrile as described in the main manuscript, the supernatant acetonitrile layer was transferred into a new tube containing 40 mg EMR (preconditioned with 250 µL water), followed by vortexing (1400 rpm, 1 min) and centrifugation (4400 G, room temperature, 5 min). The supernatant was transferred to a new test tube, and 600 µL isohexane was added for LLE, vortex (1400 rpm, 1 min), followed by another extraction step with 300 µL isohexane and evapoconcentration. In this designed workflow, compared to direct LLE after protein precipitation, the additional EMR step did not remove much extra lipids except the cholesterol eluted in the end, **Figure S3a**, but tended also to remove larger non-polar analytes that elute at later retention times (RT), **Figure S3b**.

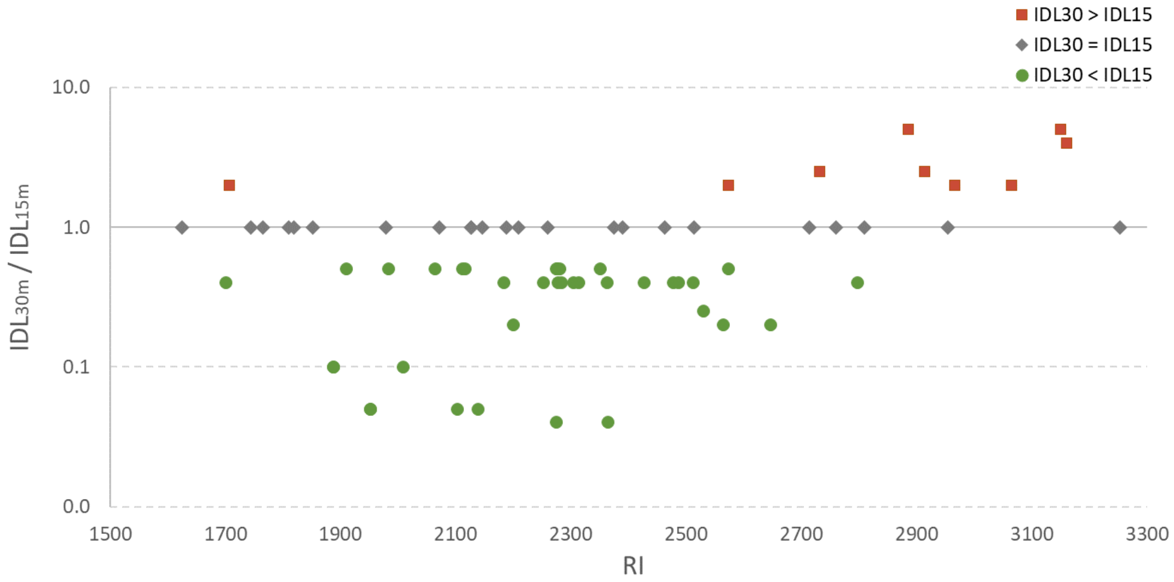

Figure S1. Ratio of instrumental detection limits (IDL) for multi-class target analytes analyzed on a 15 m and 30 m DB-5MS column using 1 µL injections. On the y-axis, values greater than 1 indicate lower detection limits (improved sensitivity) on the 15 m column, while values below 1 indicate lower detection limits (improved sensitivity) on the 30 m column. Each analyte is plotted on the x-axis according to its retention time index (RI), because of various retention times of the same analyte on the two columns. For these experiments, 11 standards (range 0.0025–50 ng/mL, triplicate) were injected on each column by programmable temperature vaporizor (PTV) in splitless mode, with injection temperature increasing from 30 to 315 °C at a rate of 7.3 °C/s. IDL was defined as the lowest injection concentration producing signal to noise ratio >3, with at least 3 data points per peak.

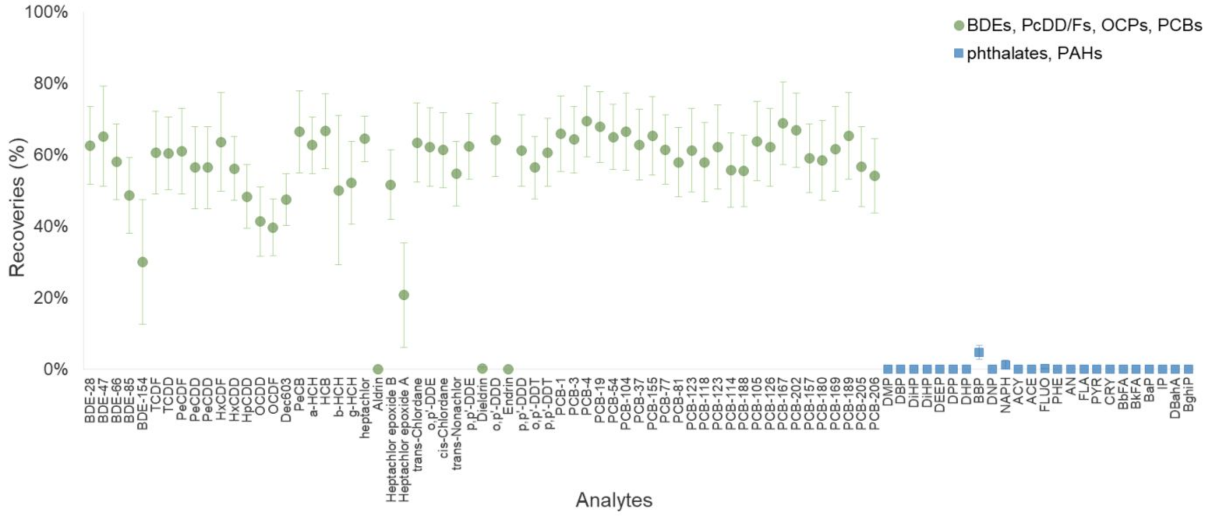

Figure S2. Absolute recoveries (n = 3) of targeted analytes in standard solution (10–13.5 ng/mL) cleaned up by acidic silica gel column. Analytes were eluted by 20 mL 10% dichloromethane in isohexane. X-axis shows the analytes sorted by chemical classes, from left to right: BDEs, PcDD/Fs, OCPs, PCBs (green) and phthalates, PAHs (blue).

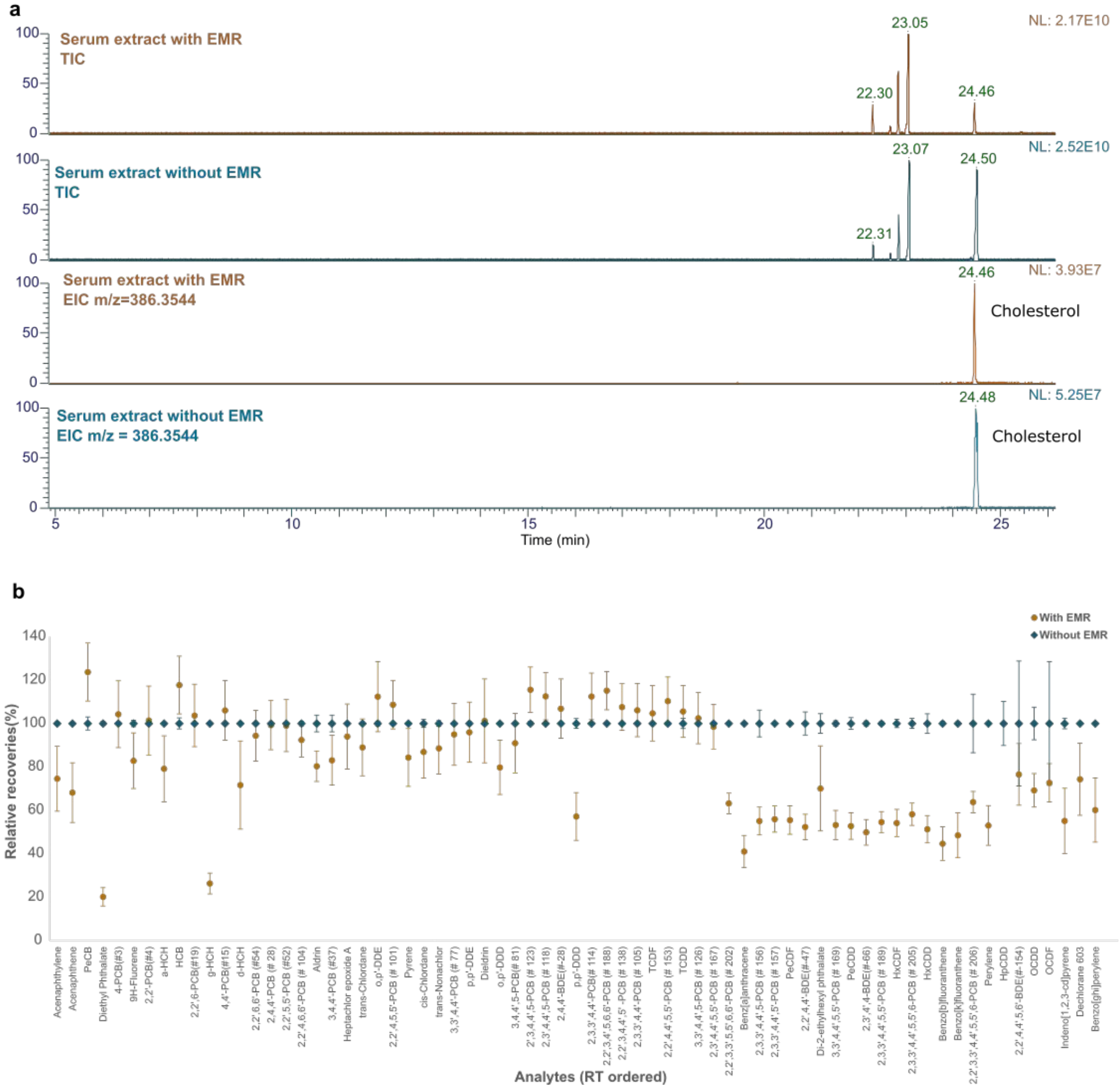

Figure S3. Chromatograms and relative recoveries of targeted analytes from 200 µL spiked commercial serum (50–67.5 ng/mL) prepared with (orange) and without (cyan) additional lipid cleanup step by Bond Elut Enhanced Matrix Removal (EMR) before liquid-liquid extraction with 600 µL and 300 µL isohexane separately. **a**. Total ion chromatograms (TIC) and extracted ion chromatograms (EIC, *m/z* = 386.3544 for cholesterol at 24.5 min) of sample extracted with and without additional EMR cleanup. The chromatograms look similar, except the last peak at 24.46 min (cholesterol) was smaller with additional EMR cleanup. While in **b**, with additional EMR cleanup, the recoveries (n = 3) of the non-polar targeted analytes (eluted in the end) were lower, compared to those without EMR (only LLE after protein precipitation). (Relative recoveries were calculated after normalizing the absolute recoveries of the same analytes to those from samples without EMR cleanup step).

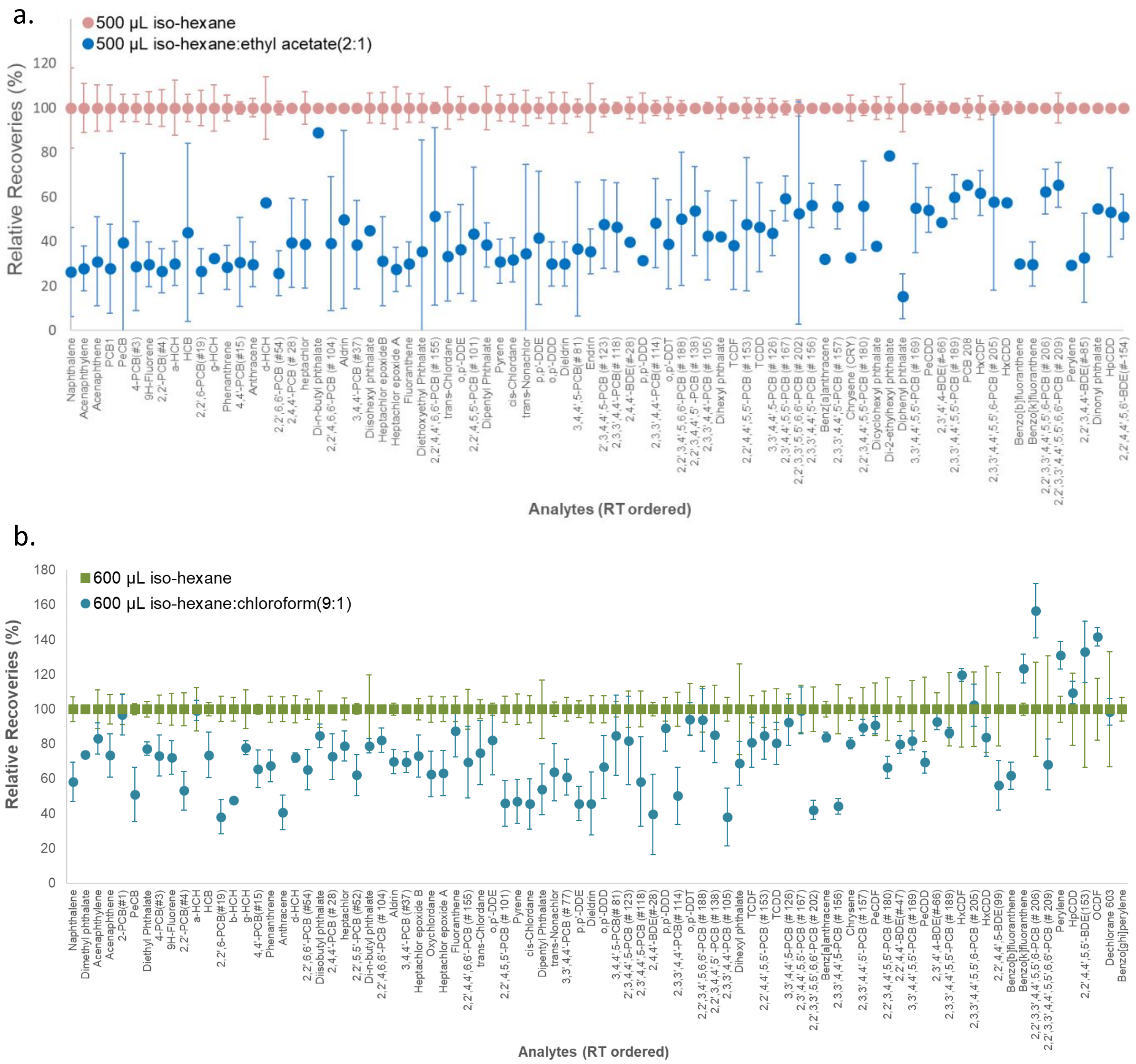

Figure S4. Relative recoveries (n = 3) of targeted analytes from spiked commercial serum extracted by pure isohexane or isohexane mixed with polar solvent. **a.** relative recoveries of analytes from 500 µL spiked commercial serum (16–28 ng/mL) extracted with 500 µL pure isohexane (pink) or isohexane: ethyl acetate (V/V = 2:1, blue) after protein precipitation with 1.5 mL acetonitrile (relative recoveries were calculated after normalizing the absolute recoveries of the same analytes to those from samples extracted with pure 500 µL isohexane). **b.** Relative recoveries of targeted analytes from 200 µL spiked serum extracted with 600 µL isohexane (in green) and isohexane: chloroform (V/V = 2:1, in blue) (relative recoveries were calculated after normalizing the absolute recoveries of the same analytes to those from samples extracted with 600 µL isohexane).

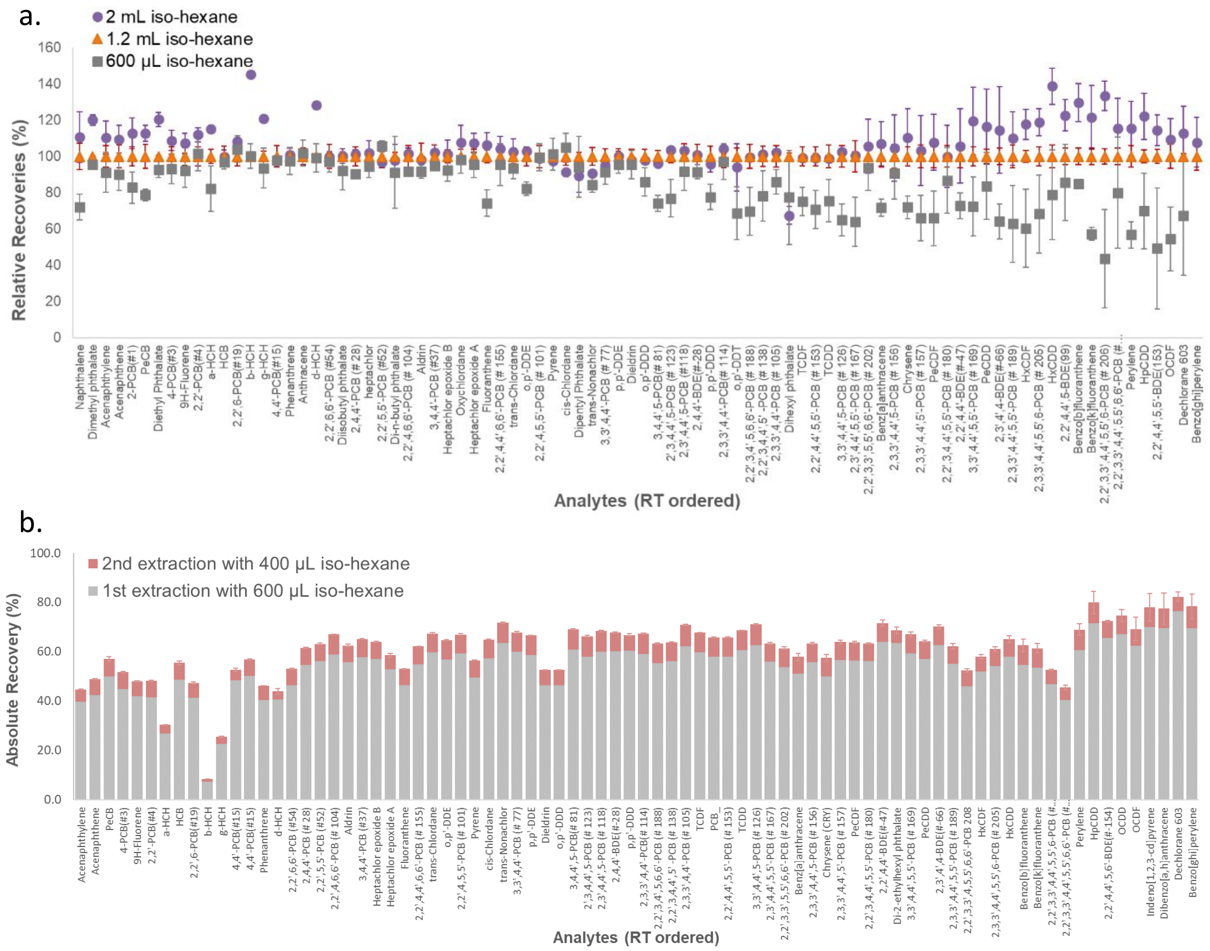

Figure S5. Recoveries (n = 3) of targeted analytes from 200 µL spiked serum (20–34 ng/mL) extracted with various volumes of isohexane (a. 600 µL in grey squares, 1.2 mL in orange triangle and 2 mL in purple circle) or additional extraction step with 400 µL isohexane (b. brown red bar). **a.** relative recoveries were calculated after normalizing the absolute recoveries of the same analytes to those from samples extracted with 1.2 mL isohexane, which was selected as the final volume, **b.** absolute recoveries of targeted analytes of first extraction with 600 µL isohexane (grey bar) and additional extraction with 400 µL isohexane.

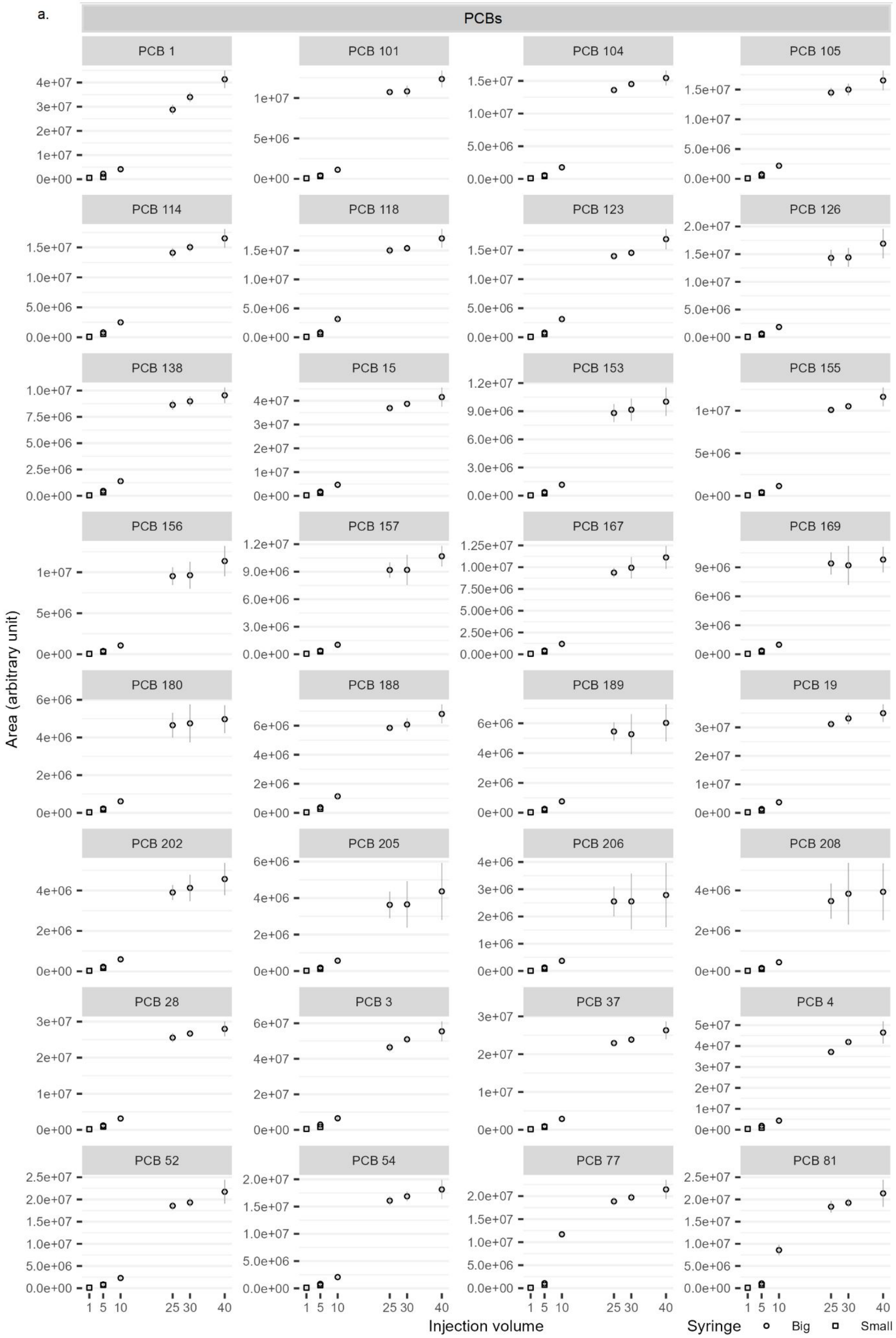
a.
PCBs
PCB 1
PCB 101
PCB 104
PCB 105
PCB 114
PCB 118
PCB 123
PCB 126
PCB 138
PCB 15
PCB 153
PCB 155
PCB 156
PCB 157
PCB 167
PCB 169
PCB 180
PCB 188
PCB 189
PCB 19
PCB 202
PCB 205
PCB 206
PCB 208
PCB 28
PCB 3
PCB 37
PCB 4
PCB 52
PCB 54
PCB 77
PCB 81
Area (arbitrary unit)
Injection volume
Syringe
Big
Small

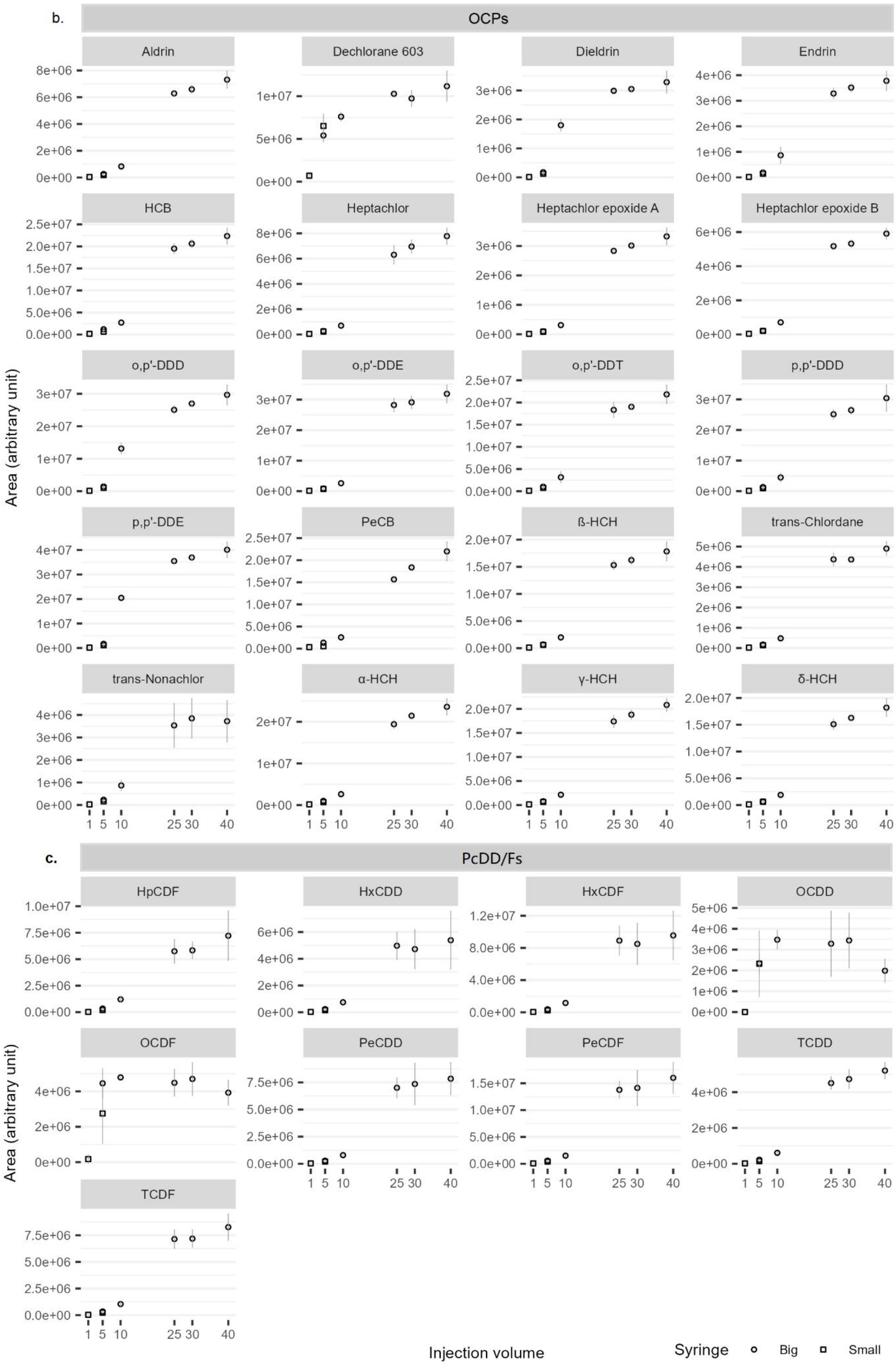
b.
OCPs
Aldrin
Dechlorane 603
Dieldrin
Endrin
HCB
Heptachlor
Heptachlor epoxide A
Heptachlor epoxide B
o,p'-DDD
o,p'-DDE
o,p'-DDT
p,p'-DDD
p,p'-DDE
PeCB
ß-HCH
trans-Chlordane
trans-Nonachlor
α-HCH
γ-HCH
δ-HCH
c.
PcDD/Fs
HpCDF
HxCDD
HxCDF
OCDD
OCDF
PeCDD
PeCDF
TCDD
TCDF
Area (arbitrary unit)
Injection volume
Syringe
Big
Small

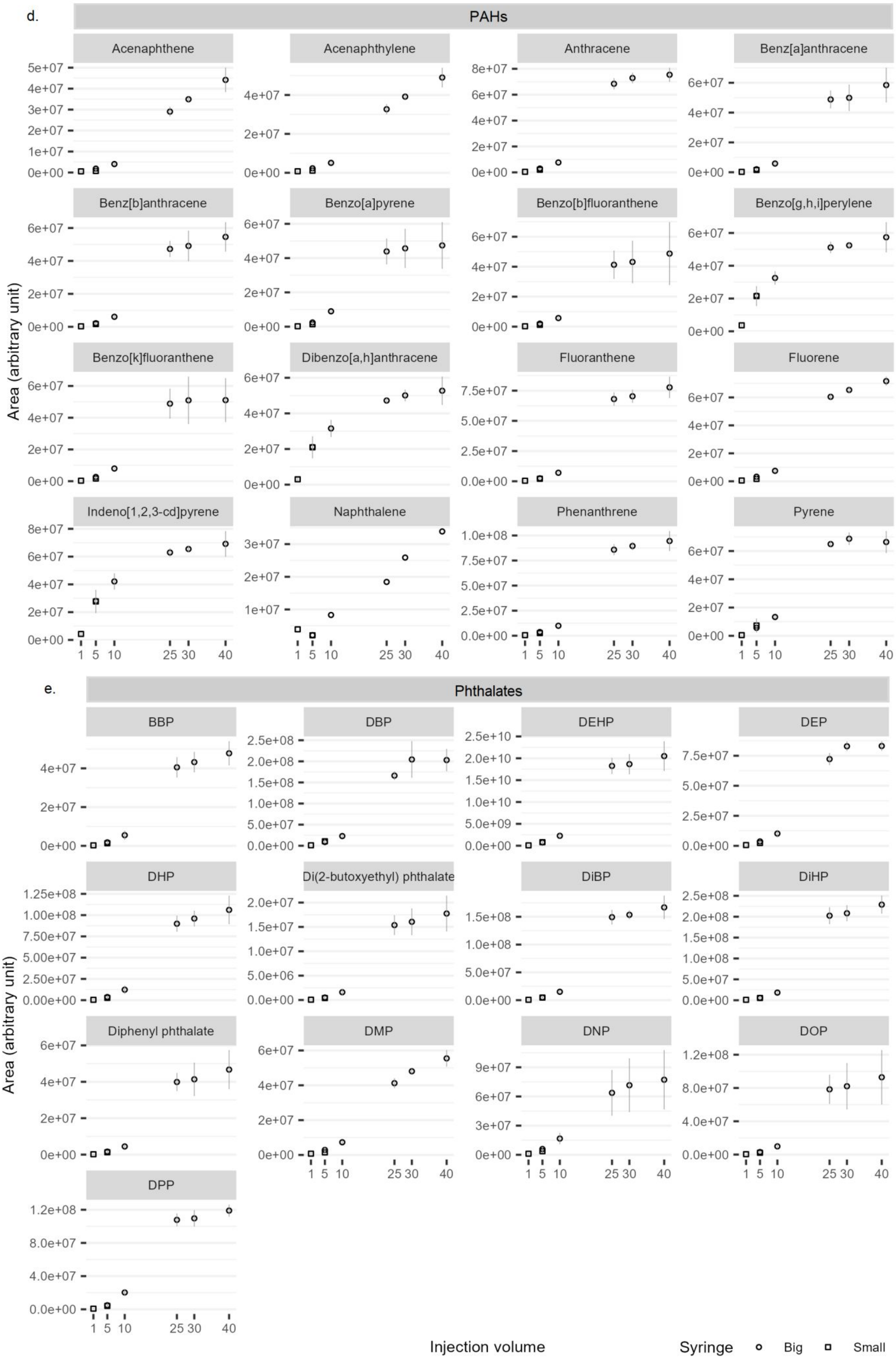
d.
PAHs
Acenaphthene
Acenaphthylene
Anthracene
Benz[a]anthracene
Benz[b]anthracene
Benzo[a]pyrene
Benzo[b]fluoranthene
Benzo[g,h,i]perylene
Benzo[k]fluoranthene
Dibenzo[a,h]anthracene
Fluoranthene
Fluorene
Indeno[1,2,3-cd]pyrene
Naphthalene
Phenanthrene
Pyrene
Area (arbitrary unit)
e.
Phthalates
BBP
DBP
DEHP
DEP
DHP
Di(2-butoxyethyl) phthalate
DiBP
DiHP
Diphenyl phthalate
DMP
DNP
DOP
DPP
Area (arbitrary unit)
1 5 10 25 30 40
Injection volume
Syringe
Big
Small

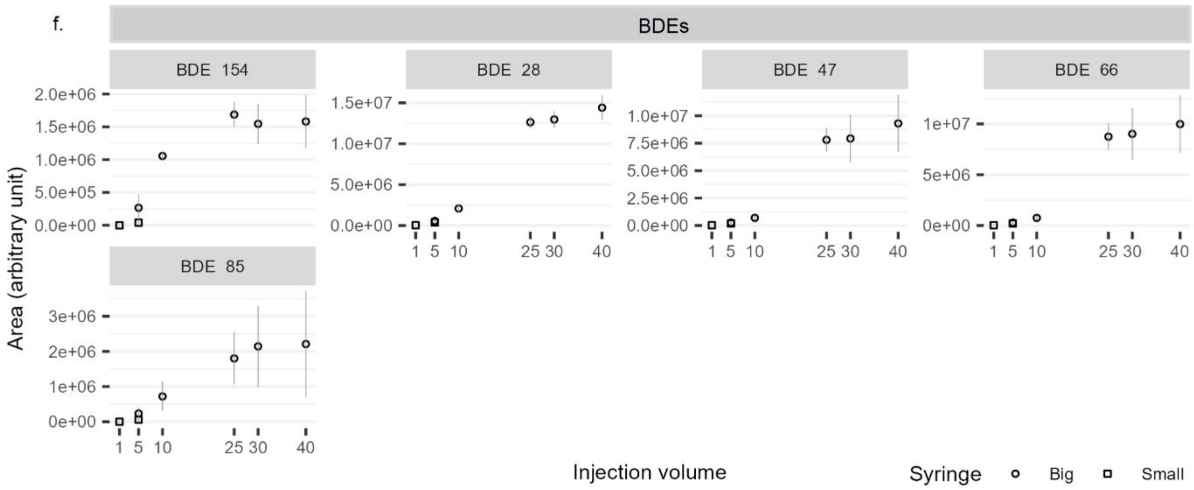

Figure S6. Increased peak areas (n ≥ 3) of the multi-class target analytes with increasing injection volumes of a spiked serum extract (10 ng/mL). Analytes from the same chemical class are plotted in each panel. X-axis shows the injection volumes and syringe used, from left to right: 1 and 5 µL with a 10 µL small syringe (square markers), and 5, 10, 25, 30, 40 µL with a 100 µL big syringe (circle markers), y axis shows the raw peak area for each analyte.

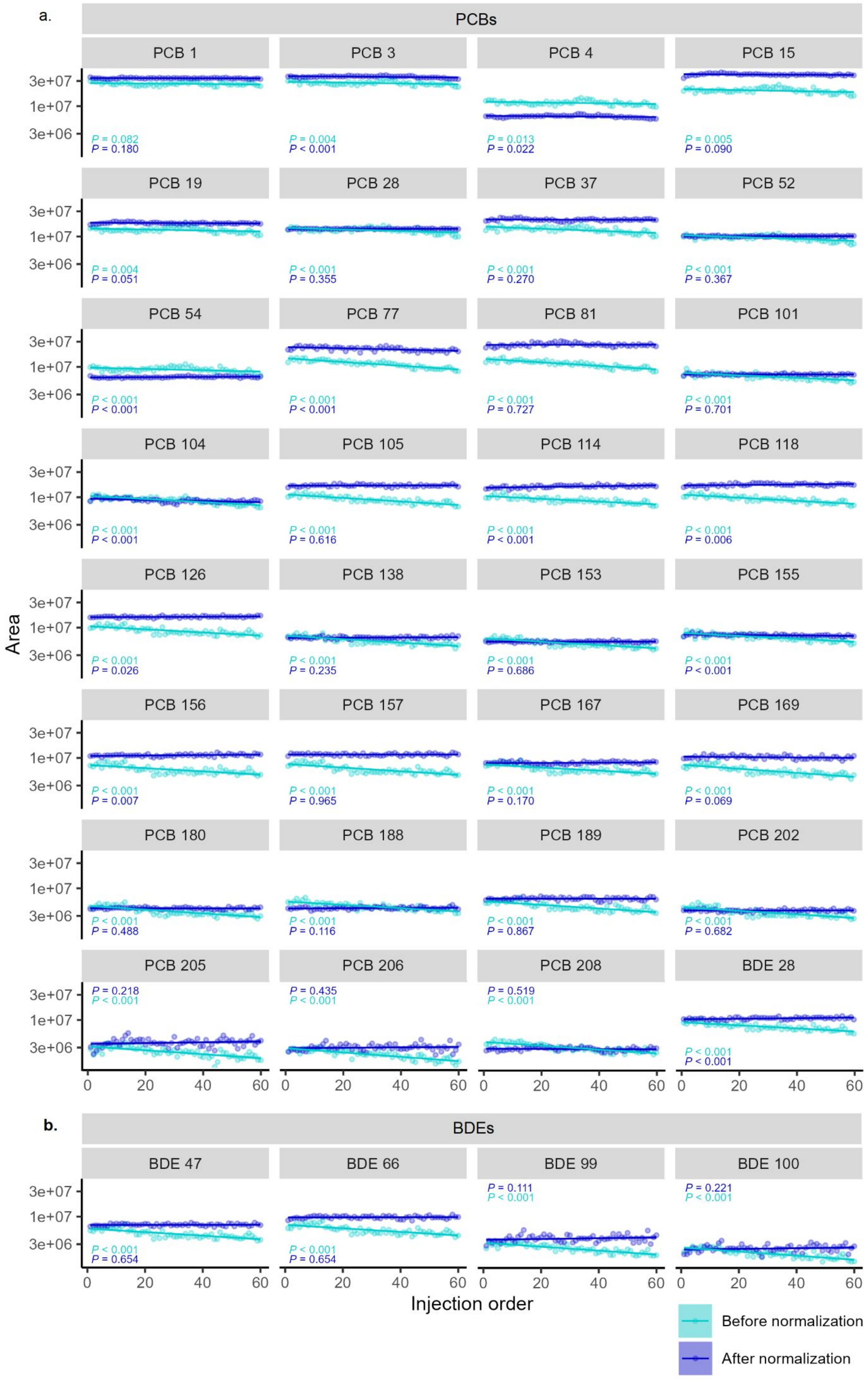

a.
PCBs
PCB 1 P = 0.082 P = 0.180
PCB 3 P = 0.004 P < 0.001
PCB 4 P = 0.013 P = 0.022
PCB 15 P = 0.005 P = 0.090
PCB 19 P = 0.004 P = 0.051
PCB 28 P < 0.001 P = 0.355
PCB 37 P < 0.001 P = 0.270
PCB 52 P < 0.001 P = 0.367
PCB 54 P < 0.001 P < 0.001
PCB 77 P < 0.001 P < 0.001
PCB 81 P < 0.001 P = 0.727
PCB 101 P < 0.001 P = 0.701
PCB 104 P < 0.001 P < 0.001
PCB 105 P < 0.001 P = 0.616
PCB 114 P < 0.001 P < 0.001
PCB 118 P < 0.001 P = 0.006
PCB 126 P < 0.001 P = 0.026
PCB 138 P < 0.001 P = 0.235
PCB 153 P < 0.001 P = 0.686
PCB 155 P < 0.001 P < 0.001
PCB 156 P < 0.001 P = 0.007
PCB 157 P < 0.001 P = 0.965
PCB 167 P < 0.001 P = 0.170
PCB 169 P < 0.001 P = 0.069
PCB 180 P < 0.001 P = 0.488
PCB 188 P < 0.001 P = 0.116
PCB 189 P < 0.001 P = 0.867
PCB 202 P < 0.001 P = 0.682
PCB 205 P = 0.218 P < 0.001
PCB 206 P = 0.435 P < 0.001
PCB 208 P = 0.519 P < 0.001
BDE 28 P < 0.001 P < 0.001
b.
BDEs
BDE 47 P < 0.001 P = 0.654
BDE 66 P < 0.001 P = 0.654
BDE 99 P = 0.111 P < 0.001
BDE 100 P = 0.221 P < 0.001
Area
3e+07
1e+07
3e+06
Injection order
0
20
40
60
Before normalization
After normalization

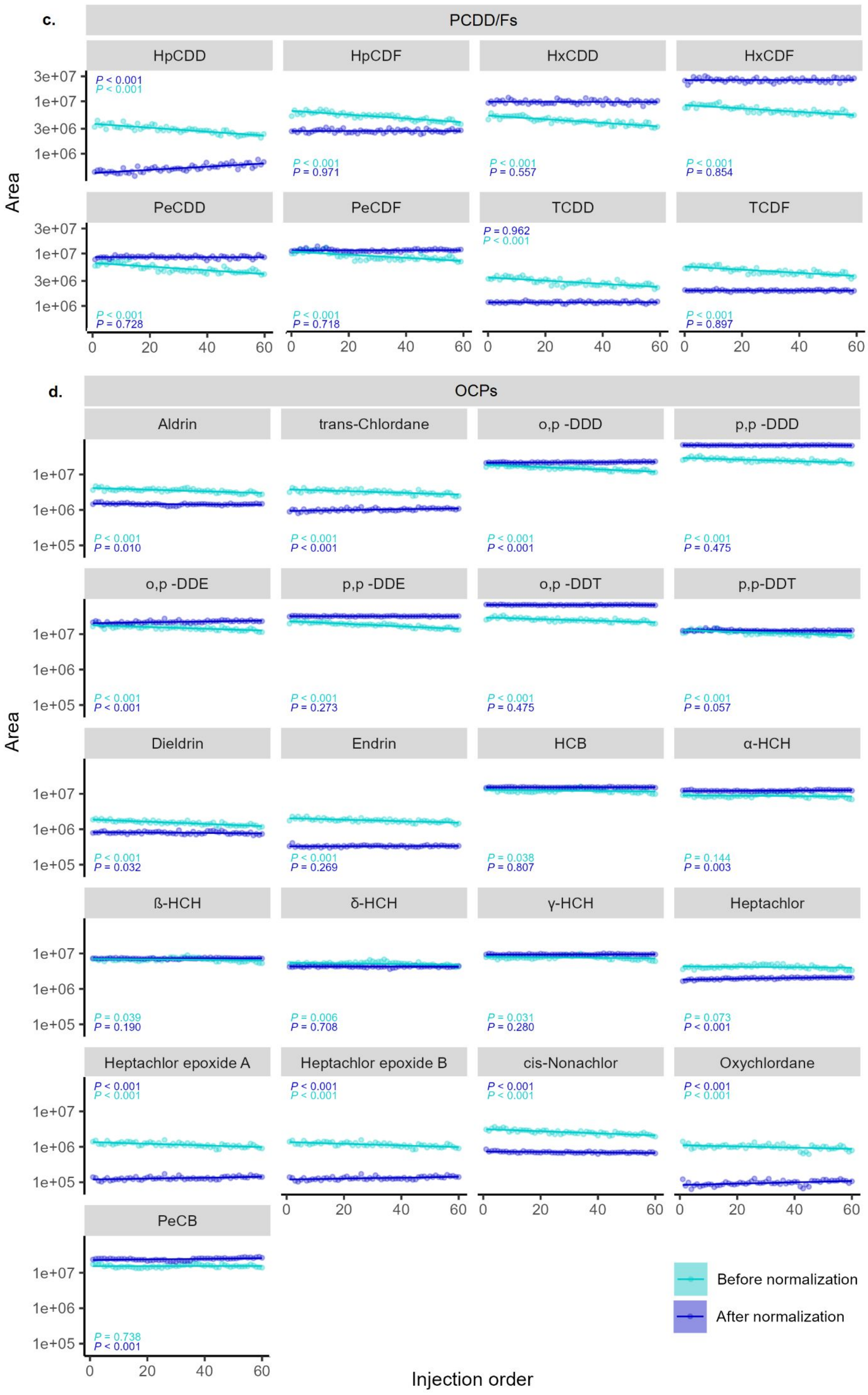
c.
PCDD/Fs
HpCDD
HpCDF
HxCDD
HxCDF
PeCDD
PeCDF
TCDD
TCDF
Area
3e+07
1e+07
3e+06
1e+06
P < 0.001
P < 0.001
P < 0.001
P = 0.971
P < 0.001
P = 0.557
P < 0.001
P = 0.854
P < 0.001
P = 0.728
P < 0.001
P = 0.718
P = 0.962
P < 0.001
P < 0.001
P = 0.897
0
20
40
60
d.
OCPs
Aldrin
trans-Chlordane
o,p -DDD
p,p -DDD
P < 0.001
P = 0.010
P < 0.001
P < 0.001
P < 0.001
P < 0.001
P < 0.001
P = 0.475
o,p -DDE
p,p -DDE
o,p -DDT
p,p-DDT
P < 0.001
P < 0.001
P < 0.001
P = 0.273
P < 0.001
P = 0.475
P < 0.001
P = 0.057
Dieldrin
Endrin
HCB
α-HCH
P < 0.001
P = 0.032
P < 0.001
P = 0.269
P = 0.038
P = 0.807
P = 0.144
P = 0.003
ß-HCH
δ-HCH
γ-HCH
Heptachlor
P = 0.039
P = 0.190
P = 0.006
P = 0.708
P = 0.031
P = 0.280
P = 0.073
P < 0.001
Heptachlor epoxide A
Heptachlor epoxide B
cis-Nonachlor
Oxychlordane
P < 0.001
P < 0.001
P < 0.001
P < 0.001
P < 0.001
P < 0.001
P < 0.001
P < 0.001
PeCB
P = 0.738
P < 0.001
Area
1e+07
1e+06
1e+05
Before normalization
After normalization
Injection order

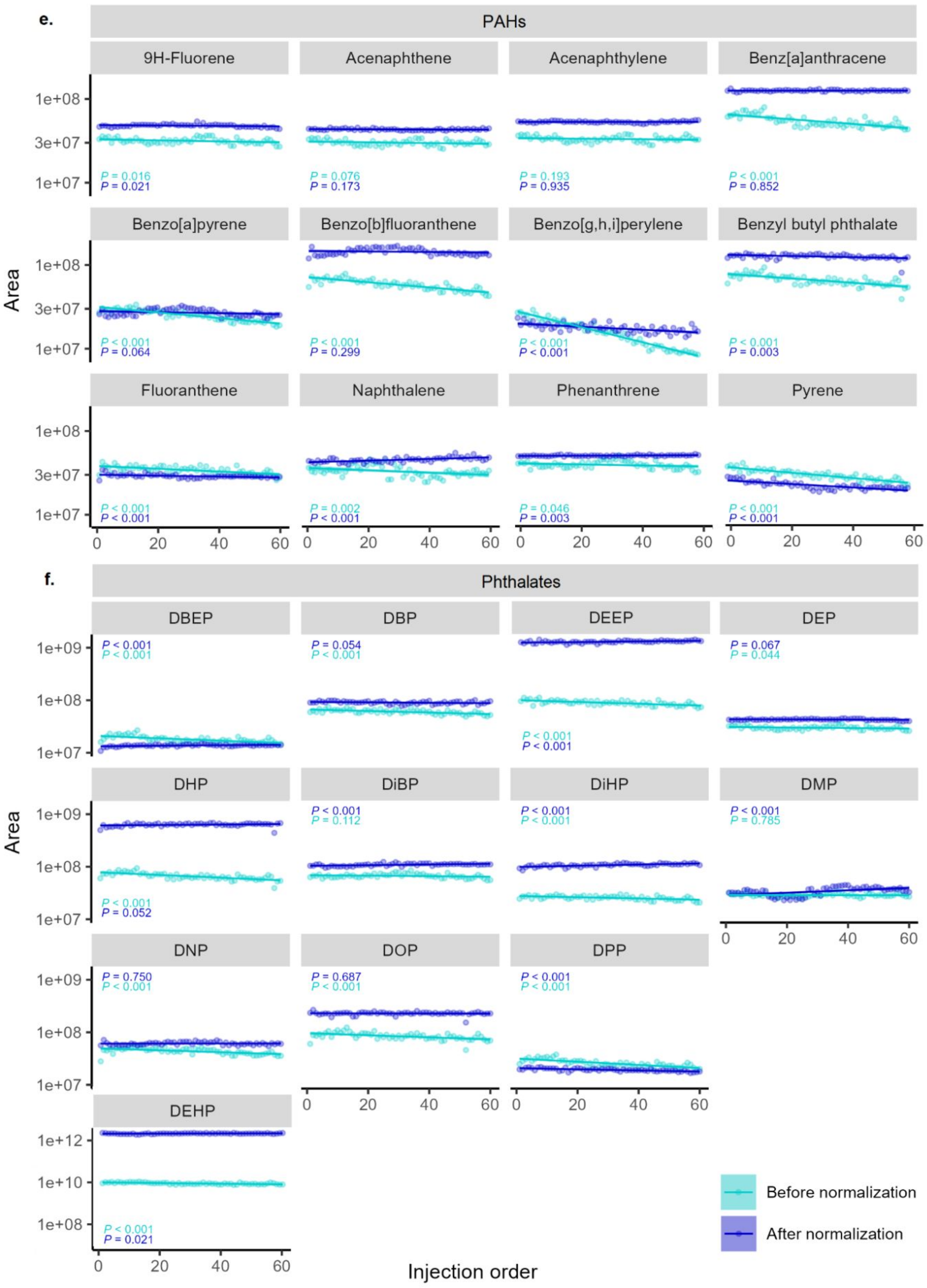

Figure S7. Responses of targeted analytes during 60 continuous 25 µL injections of spiked serum extract (2 ng/mL) before and after internal standard normalization. Analytes from the same chemical class are plotted in each panel. X-axis shows the injection order of sample extraction total there were nonstop 72 injections for 60 hrs including 12 isohexane injections. Y-axis is peak area; the light blue dots were the raw peak area of analytes; dark blue dots shows the normalized area (equation: normalized area of the analyte = (raw analyte peak area / internal standard area) * average peak area of the internal standard across all 60 injections), P value is the regression coefficient.

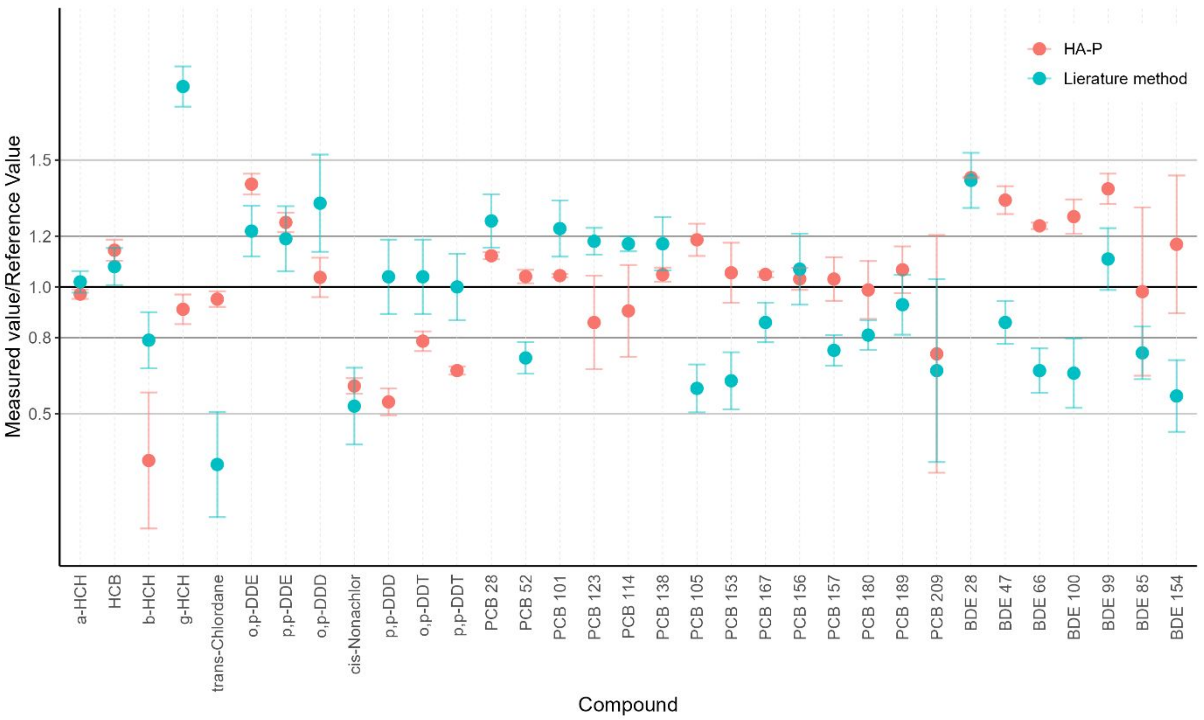

Figure S8. Quantified values by HA-P method and literature method of the standard reference materials. Ratio of quantified values to certificated reference values of 33 analytes (targeted in this study) in SRM 1958 serum by HA-P (dark orange, 25 µL injection, n = 2) and reported values by literature method[2] (cyan, n = 3) are shown.

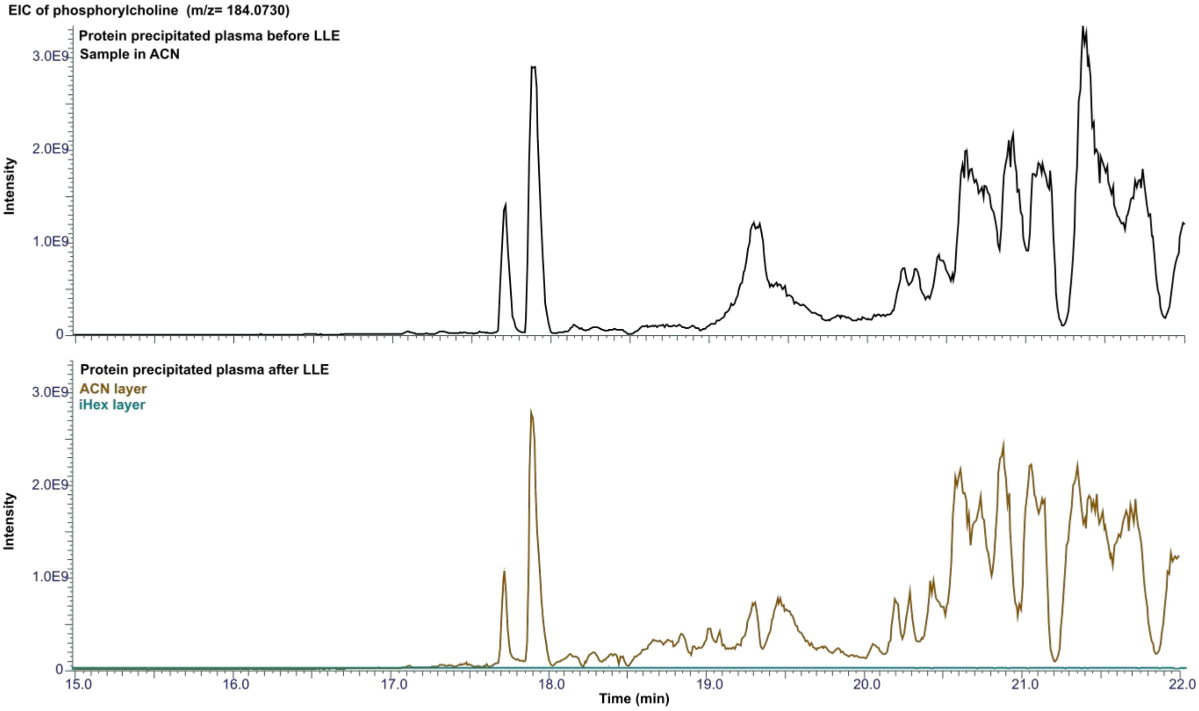

Figure S9. Extracted ion chromatograms (EIC) of phosphorylcholine fragment ion (*m/z* = 184.0730), which is a common fragment of phospholipids in LC-HRMS analysis.[4] Here we show analysis of the acetonitrile-plasma phase after protein-precipitation, before (top panel, black) and after (bottom panel, red) liquid-liquid extraction with isohexane. In the bottom panel, the isohexane layer (bottom green) was analyzed after solvent-transfer into 80% acetonitrile/20% water, demonstrating no traces of phospholipids in the isohexane extract.

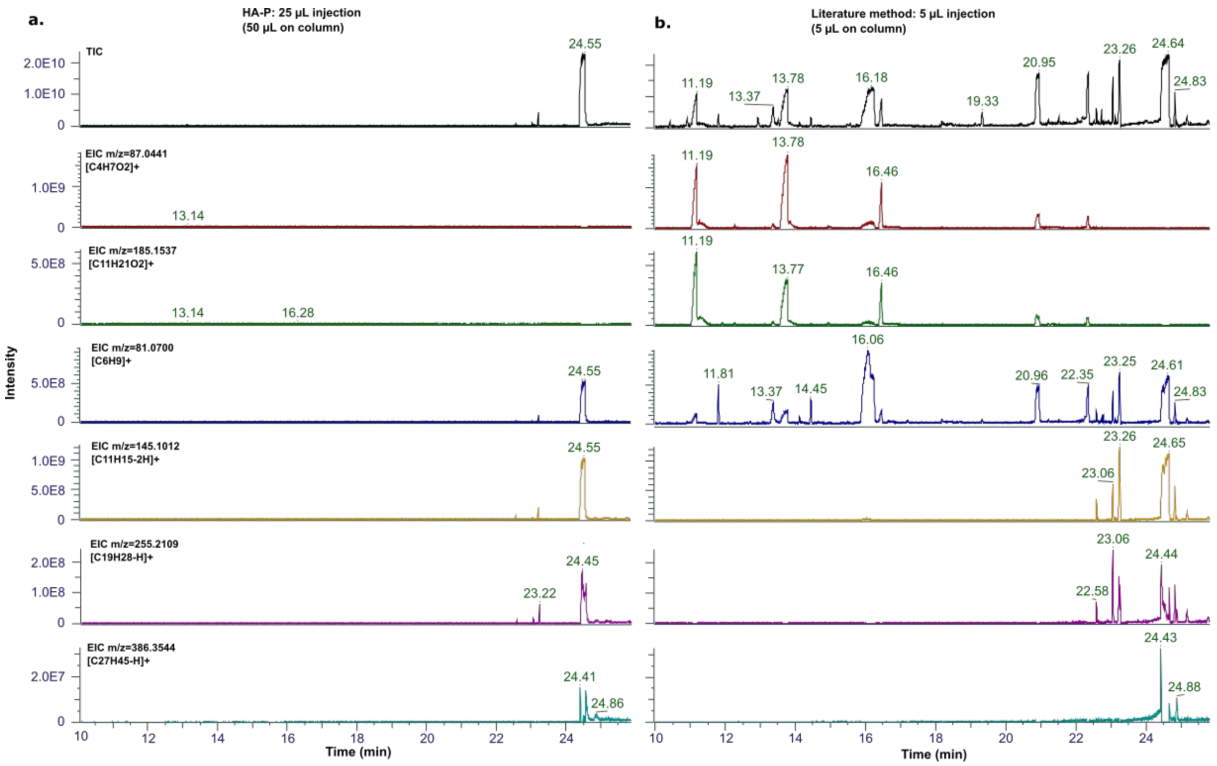

Figure S10. Extracted ion chromatograms (EIC) of long chain fatty acids and sterol lipids extracted from pooled Swedish plasma when prepared by HA-P method (a, 25 µL injection) and literature method[2] (b, 5 µL injection). From top to bottom: total ion chromatogram (TIC, black), and extracted ion chromatograms of long chain fatty acids fragments (*m/z* 87.0441 red and 185.1537 green), and EICs of sterol lipids fragments (*m/z* 81.0700 blue, 145.1012 yellow, 255.2109 pink and 386.3544 cyan), including cholesterol (i.e. cyan *m/z* 386.3544). Y-scales are the same for the left and right chromatograms.

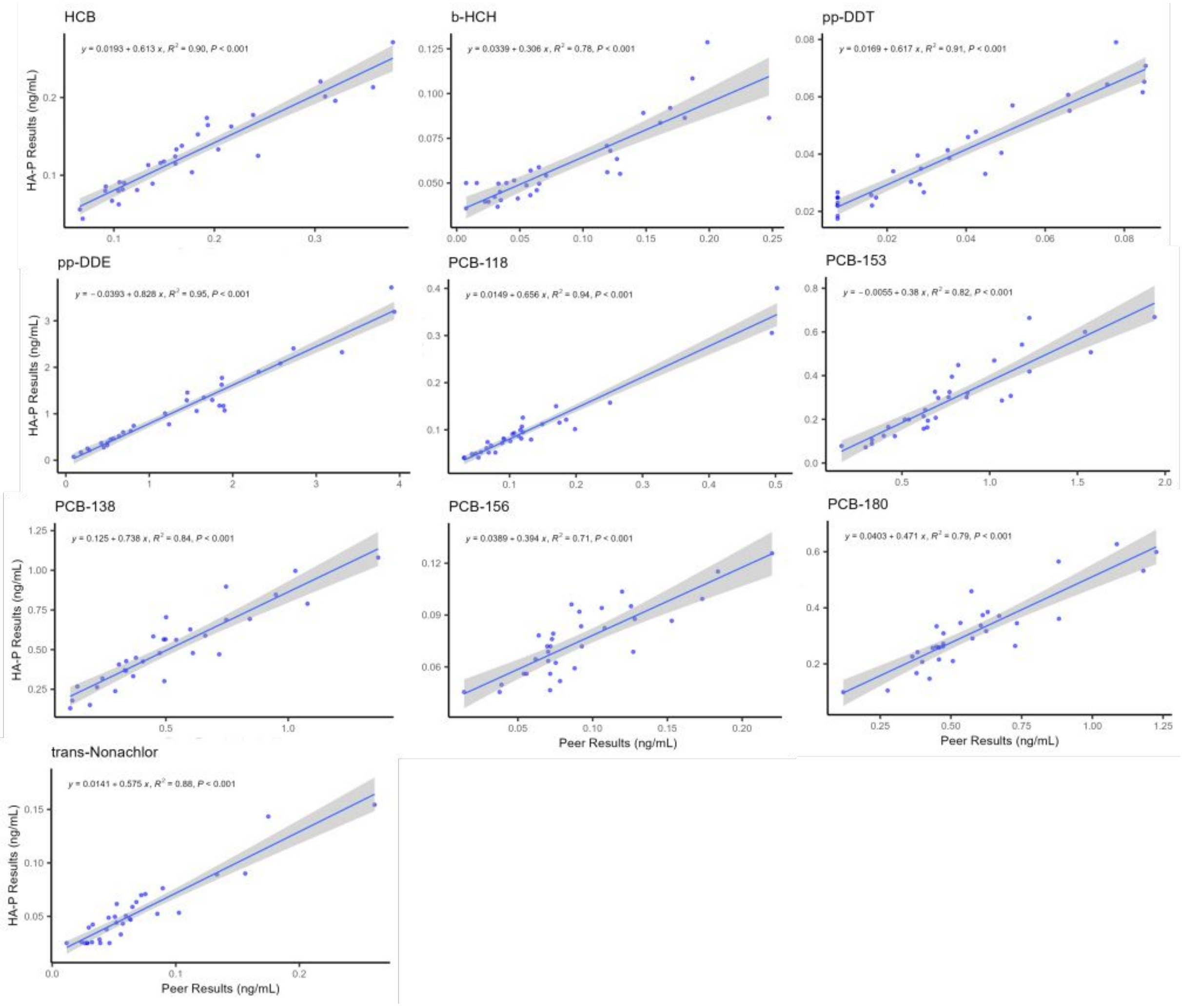

Figure S11. Quantified values by HA-P method and previous target studies of the same (different aliquots) samples. Linear regression is shown for targets concentrations by this HA-P method (y-axis) and by a peer targeted method[5] (x-axis). For each regression line, the equation, R-squared value (R^2) and p-value are reported.

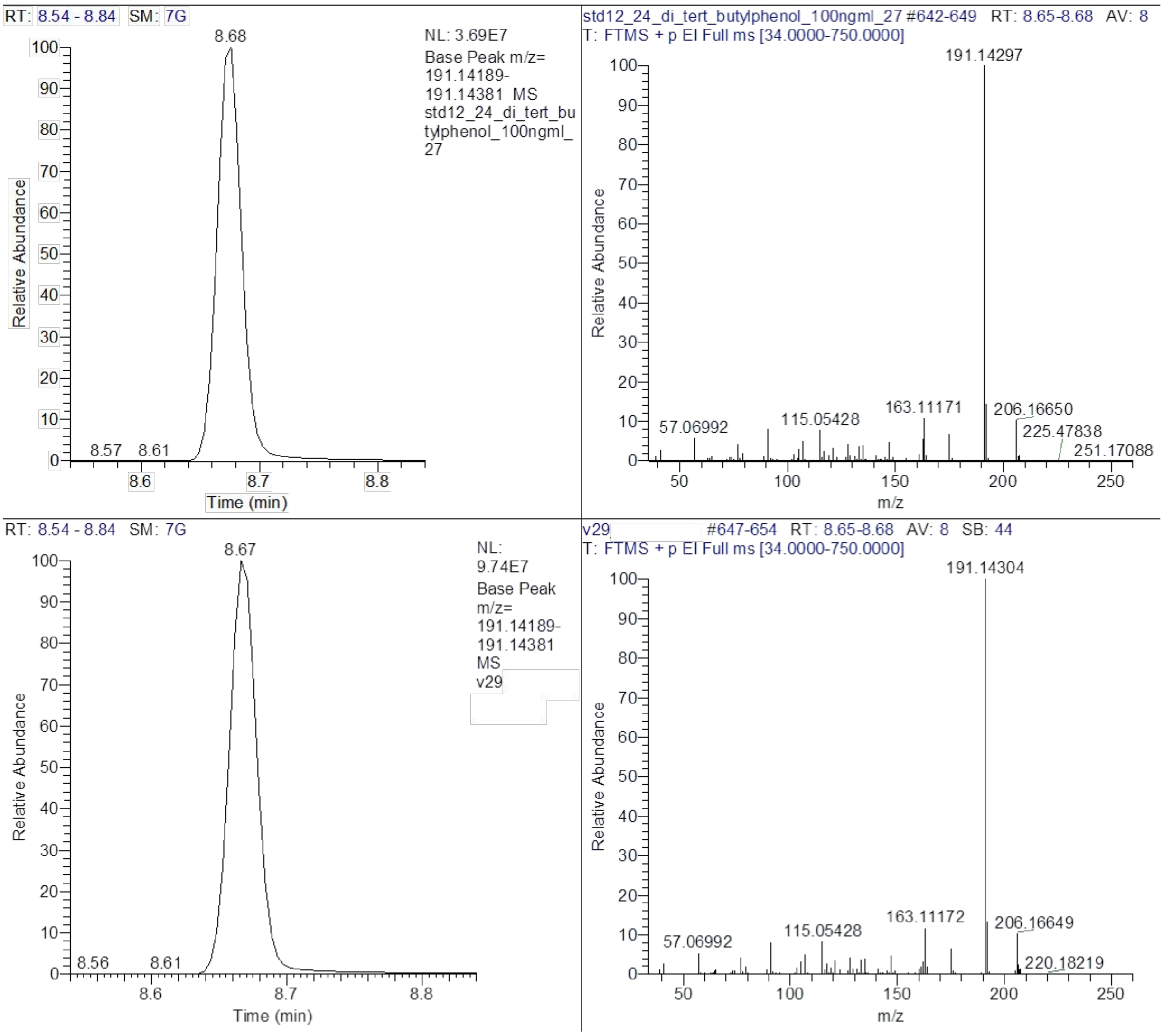

Figure S12. Extracted ion chromatogram (left) and full scan spectrum (right) for 2,4-di-tert-butylphenol in isohexane (top) and an adult plasma sample (V29, bottom).

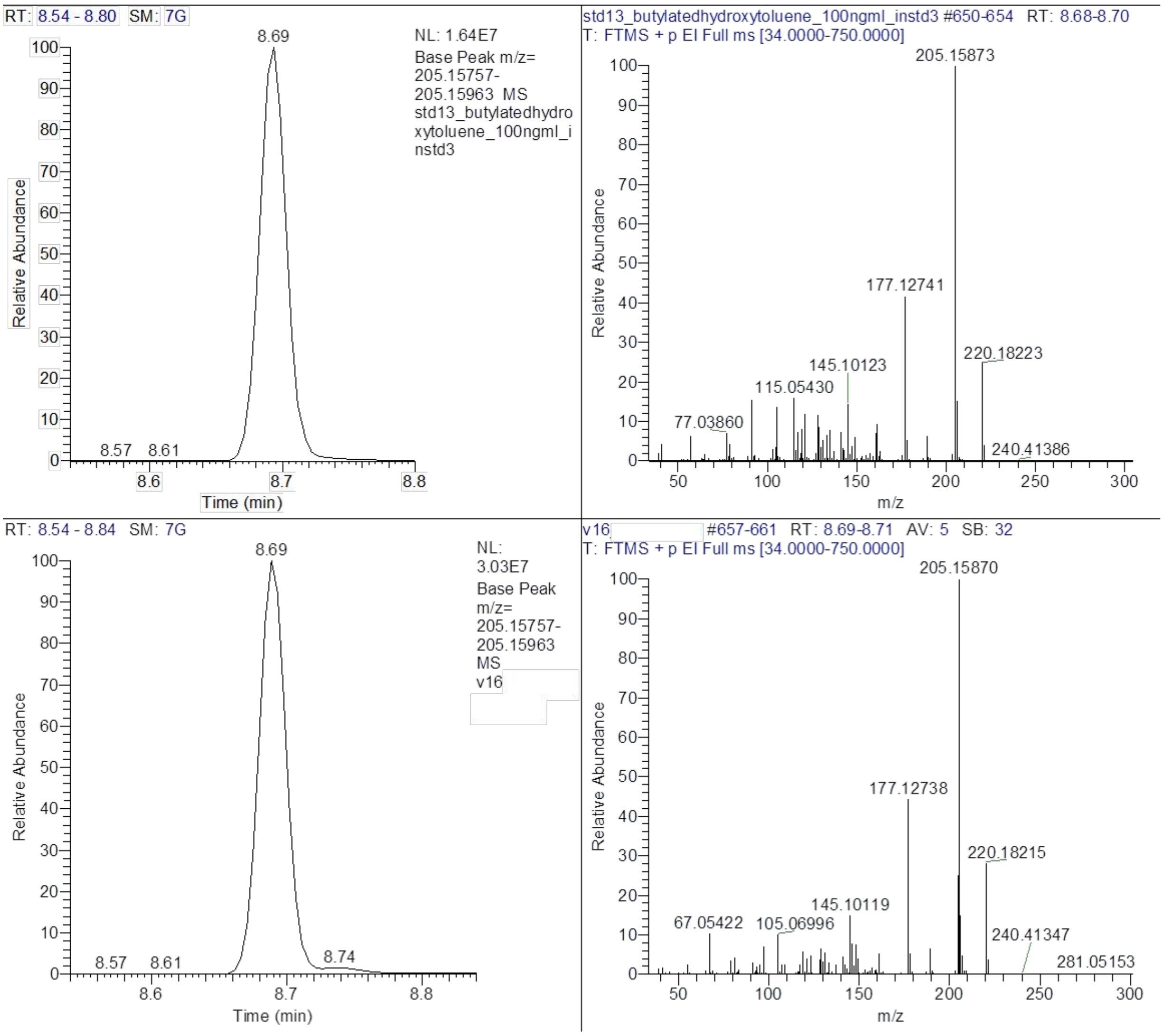

Figure S13. Extracted ion chromatogram (left) and full scan spectrum (right) for butylated hydroxytoluene in isohexane (top) and an adult plasma sample (V16, bottom).

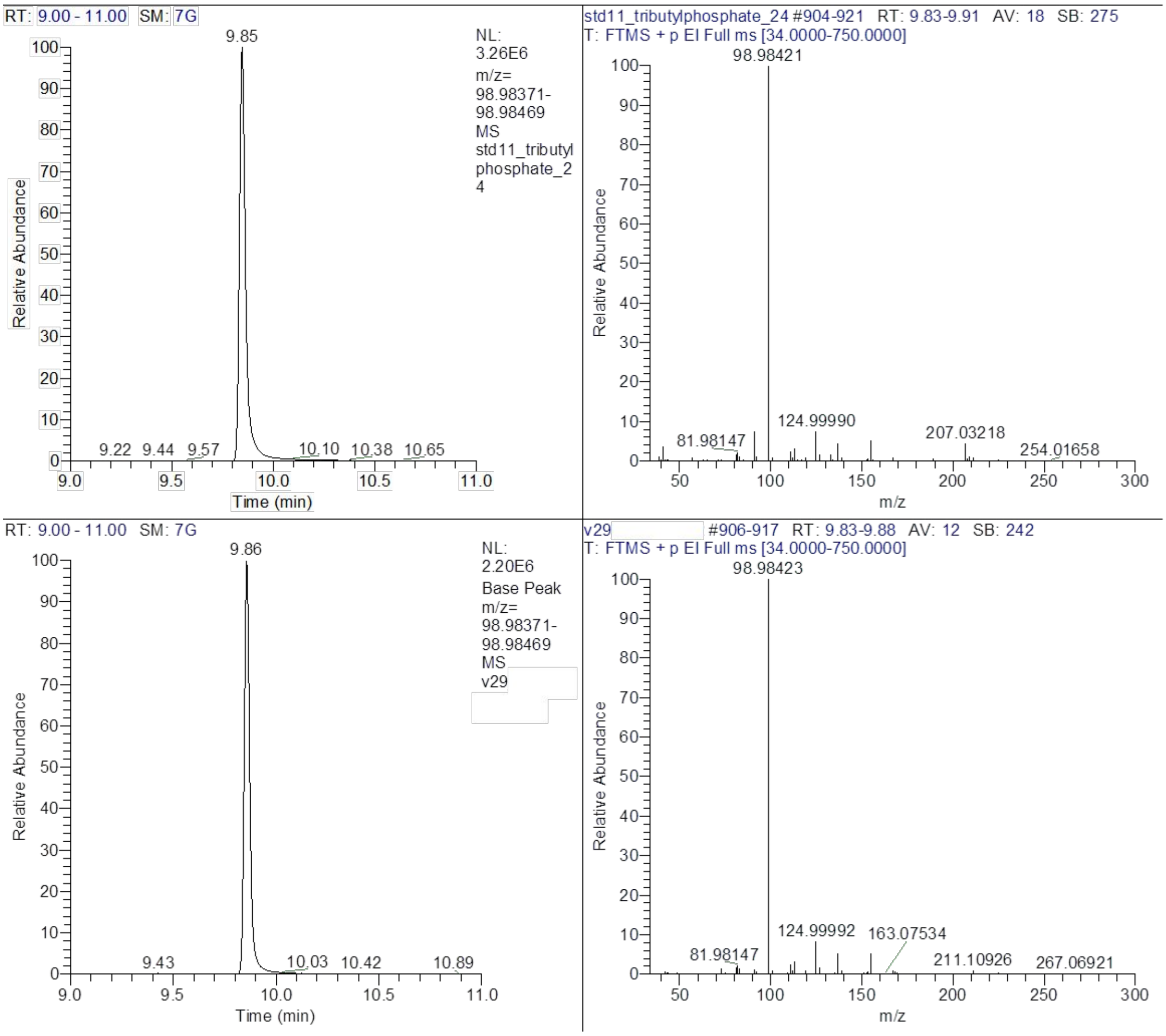

Figure S14. Extracted ion chromatogram (left) and full scan spectrum (right) for tributyl phosphate in isohexane (top) and an adult plasma sample (V29, bottom).

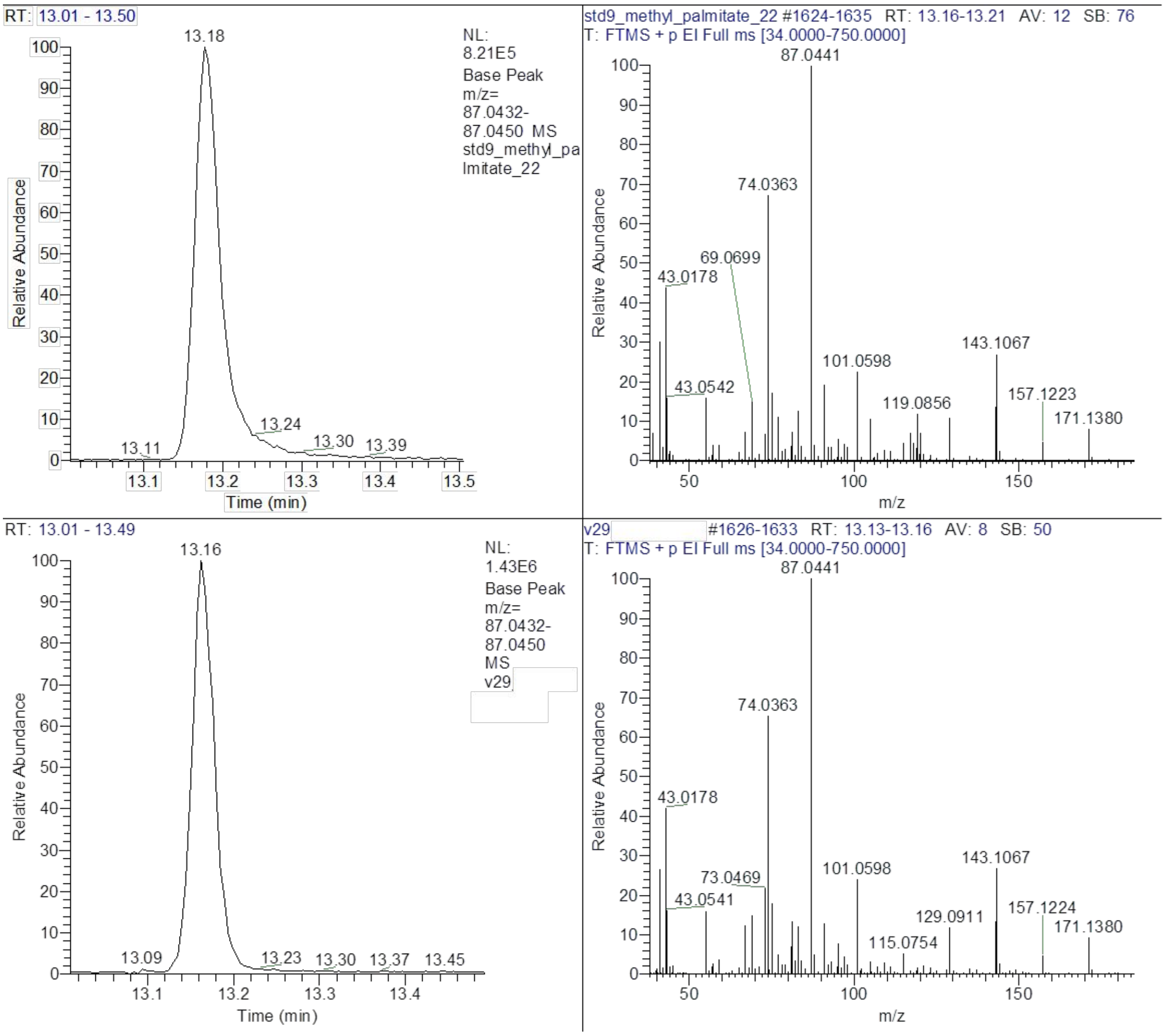

Figure S15. Extracted ion chromatogram (left) and full scan spectrum (right) for hexadecanoic acid, methyl ester (methyl palmitate) in isohexane (top) and an adult plasma sample (V29, bottom).

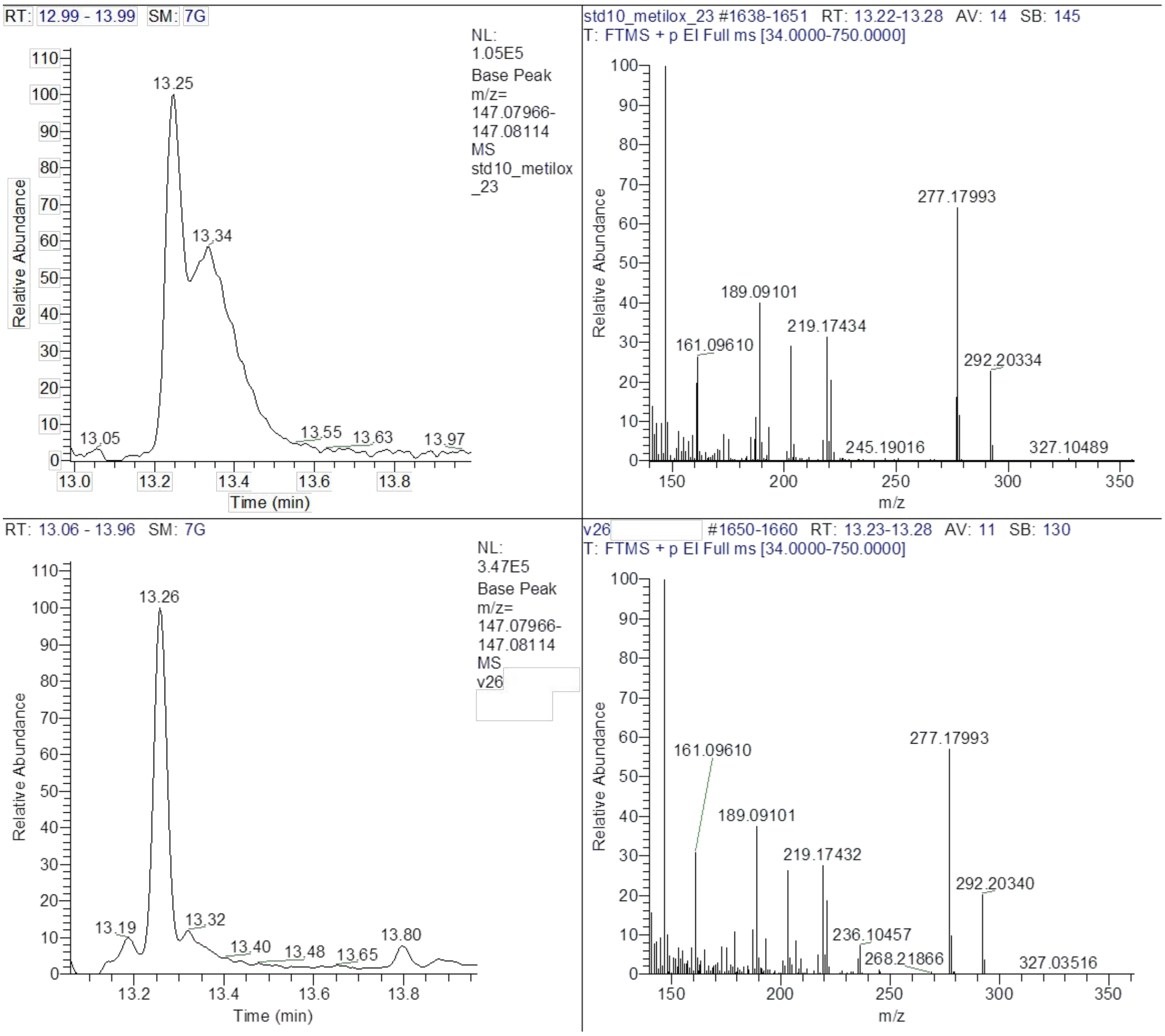

Figure S16. Extracted ion chromatogram (left) and full scan spectrum (right) for methyl-3-[3,5-di(tert-butyl)-4-hydroxyphenyl] propanoate (metilox) in isohexane (top) and an adult plasma sample (V26, bottom). Metilox is air sensitive and prone to degradation,[6,7] the shoulder peak in the standard injection (top left) is probably degradation products of metilox before or during analysis.

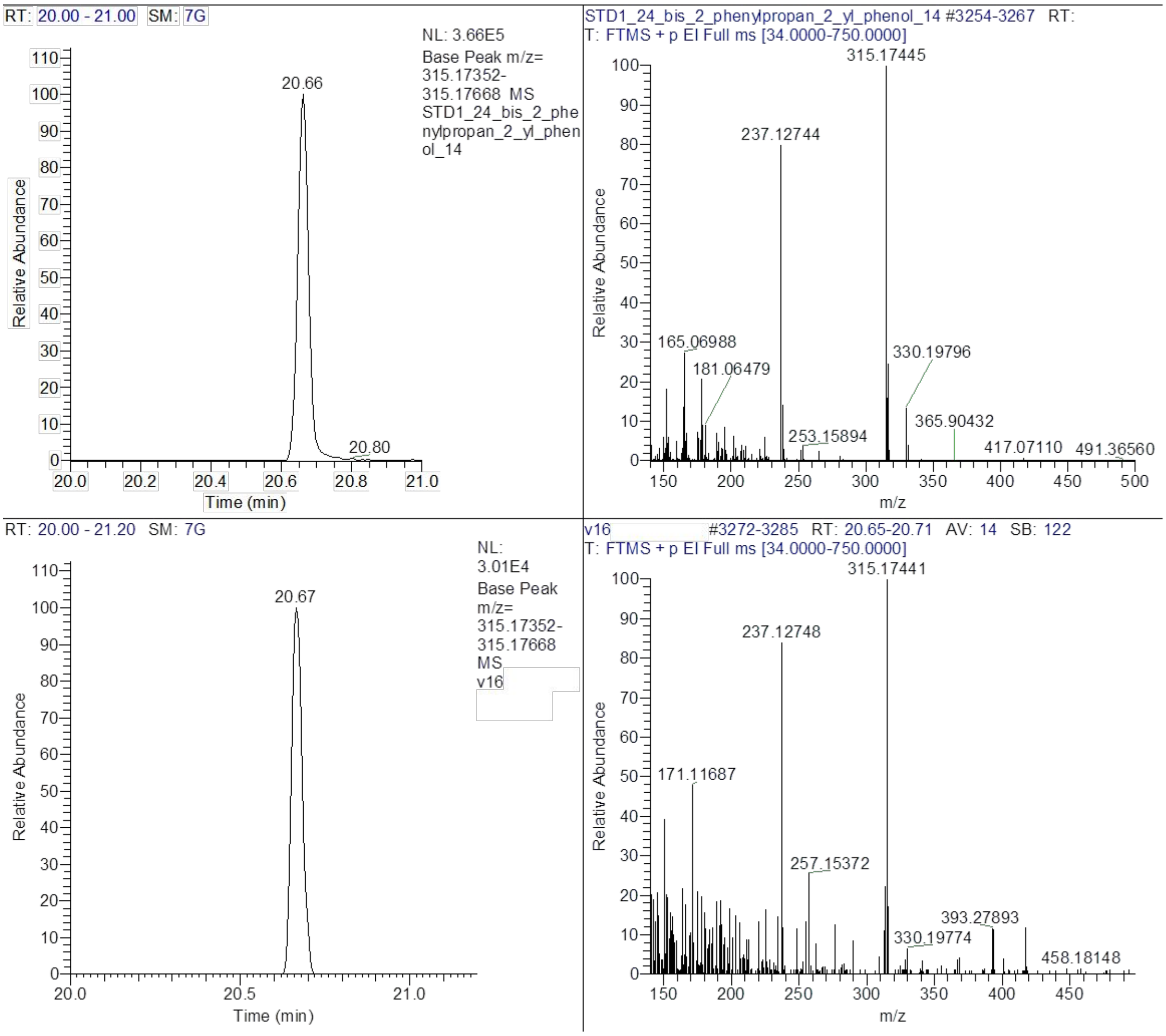

Figure S17. Extracted ion chromatogram (left) and full scan spectrum (right) for phenol, 2,4-bis(1-methyl-1-phenylethyl)- (2,4-bis(2-phenylpropan-2-yl) phenol) in isohexane (top) and an adult plasma sample (V16, bottom).

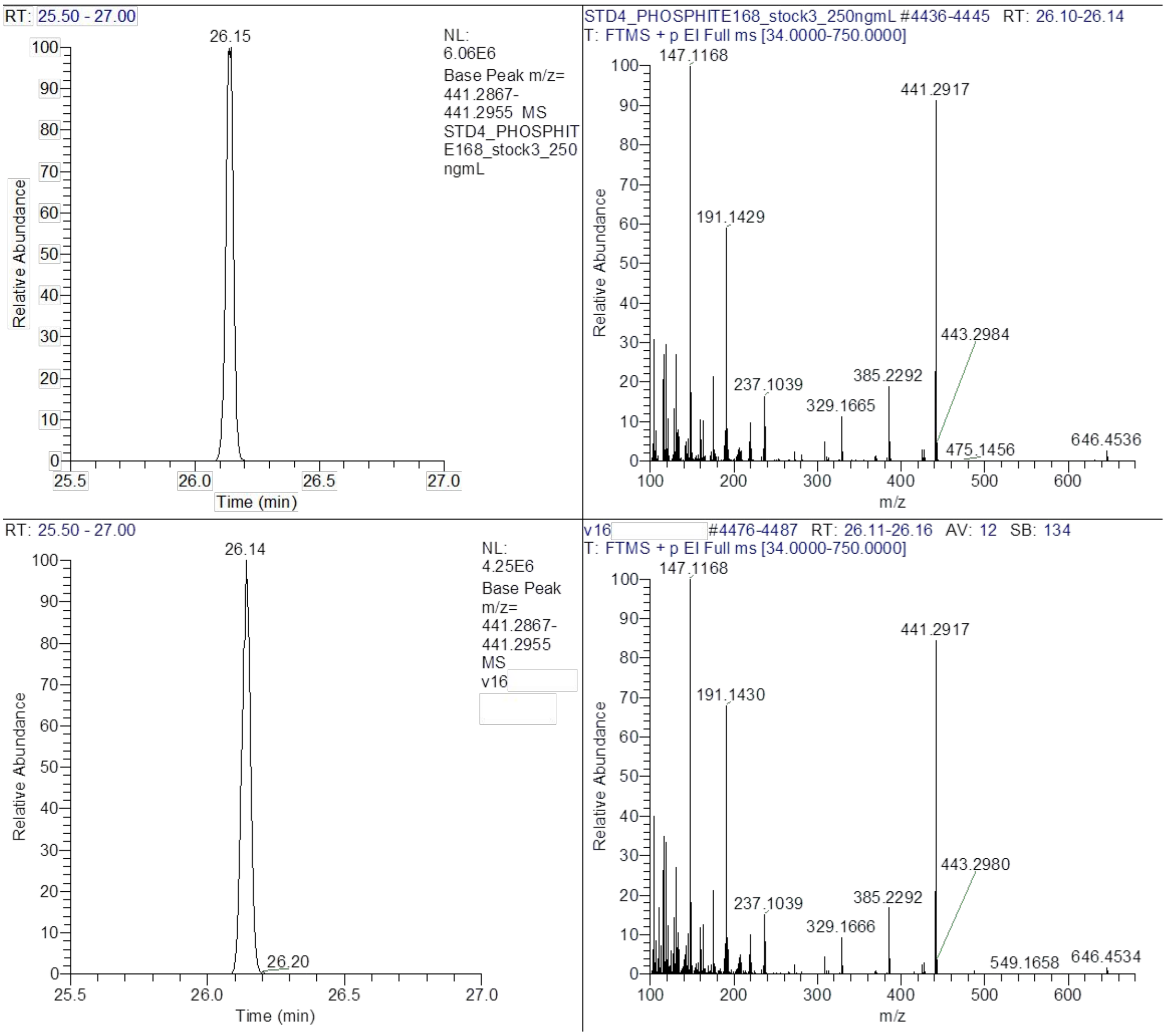

Figure S18. Extracted ion chromatogram (left) and full scan spectrum (right) for tris(2,4-di-tert-butylphenyl) phosphite (Phosphite 168) in isohexane (top) and an adult plasma sample (V16, bottom).

LITERATURE CITED

1. Andreas Sjodin. *Serum Isotope dilution High resolution Mass Spectrometry (IDHR-MS). CDC NHANES 2015-2016 Laboratory Procedure Manual* (2016).

2. Hu, X. *et al.* A scalable workflow to characterize the human exposome. *Nat. Commun. 2021 121* **12**, 1–12 (2021).

3. S, L. *et al.* Magnetic metal-organic frameworks coated stir bar sorptive extraction coupled with GC-MS for determination of polychlorinated biphenyls in fish samples. *Talanta* **144**, 1139–1145 (2015).

4. Sdougkou, K. *et al.* Phospholipid Removal for Enhanced Chemical Exposomics in Human Plasma. *Environ. Sci. Technol.* **57**, 10173–10184 (2023).

5. Donat-Vargas, C. *et al.* Persistent organochlorine pollutants in plasma, blood pressure, and hypertension in a longitudinal study. *Hypertension* **71**, 1258–1268 (2018).

6. ECHA CHEM. Reaction mass of isomers of: C7-9-alkyl 3-(3,5-di-tert-butyl-4-hydroxyphenyl)propionate. Available at: https://echa.europa.eu/registration-dossier/-/registered-dossier/8689/5/1. (Accessed: 5th March 2024)

7. Blázquez-Blázquez, E., Cerrada, M. L., Benavente, R. & Pérez, E. Identification of Additives in Polypropylene and Their Degradation under Solar Exposure Studied by Gas Chromatography-Mass Spectrometry. *ACS Omega* **5**, 9055–9063 (2020).
